# Trend analysis of reaction parameters and properties of plant-mediated green synthesized selenium nanoparticles using non-parametric statistical methods (Mann–Kendall trend test, Sen's slope estimator, ANOVA) and their applications in the modern world: a critical perspective

**DOI:** 10.1039/d5ra03940a

**Published:** 2025-08-07

**Authors:** Md. Saiful Islam, Sams Uddin Sams, Sadit Bihongo Malitha, Md. Zahangir Alam

**Affiliations:** a Department of Applied Chemistry and Chemical Engineering, Faculty of Engineering and Technology, University of Dhaka Dhaka 1000 Bangladesh zahangir@du.ac.bd; b Atish Dipankar University of Science & Technology Dhaka 1230 Bangladesh

## Abstract

Selenium nanoparticles (SeNPs) have garnered increased interest due to their significant role in human health, including the regulation of blood sugar levels, boosting the immune system, reducing oxidative stress, and other benefits. Conventional synthesis methods of SeNp employ the use of toxic chemicals and solvents, which are harmful to the environment; in contrast, the green synthesis method does not involve the use of any toxic chemicals. In this article, the green synthesis of SeNp using plant extracts, the influence of reaction parameters on their characteristic properties, and the application of SeNp in the present world are discussed. The temperature and stirring speed showed a significant correlation with the particle size and zeta potential, whereby increasing either of these reaction parameters resulted in a decrease in the characteristic properties mentioned. To further confirm the correlation, the Mann–Kendall Trend Test was performed, which confirmed the decreasing trend of particle size and zeta potential with increasing temperature and stirring speed. The volume and concentration of precursor solution and plant extract gave no significant correlation, as different plant extracts comprise different chemical compositions. The polydispersity index (PDI) showed no correlation with any of the reaction parameters due to a lack of adequate data in the literature. Green-synthesised SeNp, owing to its anti-cancer, antioxidant, anti-diabetic, and many other biomedically significant properties, is currently emerging as an important material in biomedical applications. They also have applications in electronics, catalysis, and sensors, with significant positive impacts on crops and aquaculture, which are thoroughly discussed in this article.

## Abstract 2

Selenium nanoparticles (SeNPs) have garnered increased interest due to their significant role in human health, including the regulation of blood sugar levels, boosting the immune system, reducing oxidative stress, and other benefits.

## Graphical abstract

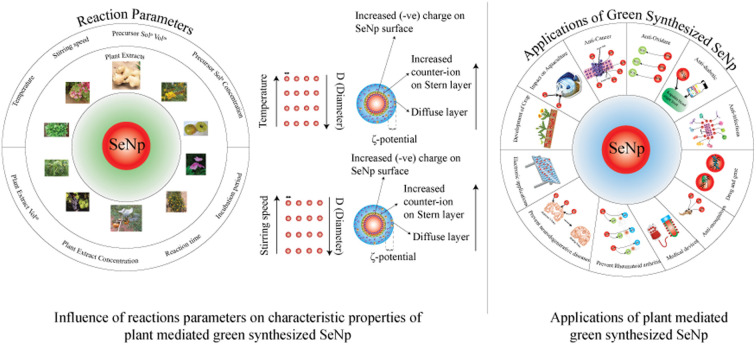

## Introduction

Metal nanoparticles, owing to their unique properties such as high surface area, high reactivity, and distinctive optical and electronic properties, have garnered the attention of researchers in the present day.^1^ Selenium is naturally scattered throughout the Earth's crust but finds extensive use owing to its excellent semiconducting and photoelectric sensing properties.^2^ Naturally, two forms of selenium are found in nature: inorganic (selenite and selenate) and organic (selenomethionine and selenocysteine).^3^ The core structure of selenium is complex, leading to four different isomers: amorphous selenium (α – Se), (α – monoclinic), β – monoclinic and hexagonal crystalline selenium (most stable).^4,5^ Between monoclinic and trigonal forms, monoclinic and trigonal are crystalline (t-Se is more stable), and the m-Se shows the colour red, while t-Se shows the colour black. Amorphous selenium is also red. In the nanoregime, selenium particles exhibit enhanced efficiency in their applications due to their higher surface-to-volume ratio.^3^ Selenium nanoparticles have attracted growing interest due to their wide variety of applications. It plays an important role in human health, for example, by influencing thyroid hormone metabolism and immune function.^6^ Selenium works as an antioxidant because it has a key role as a part of glutathione peroxidase, which is an enzyme working as a protector of essential SH-groups and also performs the decomposition of peroxides. It has a bactericidal action, as it can cause the death of microscopic organisms by catalysing the oxidation of intracellular thiols.^7–9^ Various articles in the literature support their anticancer, antioxidant, antimicrobial, and antibiofilm properties, as well as their anti-hydroxyl radical, chemopreventive, and DNA oxidation preventive properties.^10–18^ Besides biomedical sectors, selenium nanoparticles also have a wide range of applications. They play a key role in renewable energy devices, according to a previous study.^19^ SeNp plays a key role in protecting the environment by capturing the heavy and toxic metal mercury, which is highly detrimental to the environment.^20^ It can be used as an elemental semiconductor, used in the production of photovoltaic cells, rectifiers, photographic exposure meters, xerography, and so on.^21^

Three types of methods are employed in the synthesis of selenium nanoparticles, namely chemical, physical, and biological.^3^

For the synthesis of SeNp with a physical approach, pulsed laser ablation,^22–24^ vapor deposition,^25^ hydrothermal,^26^ and solvothermal methods^27^ are commonly employed. Bacteria and fungi, as well as enzyme-mediated synthesis of SeNp, are reported in the literature, which falls under the category of biological methods.^1^ The chemical method mainly comprises the reduction of the SeNp from the selenium salt, namely sodium selenite, sodium selenate, sodium selenosulphate, selenious acid, and selenium oxide. For the chemical reduction, use of reducing agents such as ascorbic acid,^28^ glucose,^28^ starch,^28^ fructose,^29^ cysteine,^30^ glutathione,^31^ and ionic liquid 1-ethyl-3-methyl-imidazolium thiocyanate^6^ is reported in the previous literature. The chemicals used as the reducing agent in the conventional synthesis may be toxic, and in some cases, costly, and environmentally harmful too. Thus, despite the other methods ascribed, the green synthesis of selenium nanoparticles using plant extracts is widely employed due to its low toxicity, minimal use of hazardous materials, wide range of applicability, and low toxicity for biomedical applications.^32^

The operational parameters influence the characteristics of the resulting selenium nanoparticles. The precursor salt solution, its concentration, reaction temperature, plant extract concentration, stirring speed, and all these input parameters have a significant impact on the nanoparticle size, hydrodynamic diameter, morphology, zeta potential, polydispersity index, and crystallinity index of the resulting nanoparticle. As reported in previous literature, as the temperature increases, the nanoparticle size decreases. With an increase in reaction time, the resulting particles may either form aggregates or shrink in size.^1^ However, to the best of our knowledge, the correlation between the temperature of the reaction medium and zeta-potential and the correlation between stirring speed and particle size and zeta-potential have not been reported yet in the literature. Very few works have discussed the relationship between process parameters and the characteristic properties of nanoparticles,^33–36^ but no such work has been reported for selenium nanoparticles. The existing works did not provide any dataset or statistical analysis to devise the correlation.

In this article, the correlation between reaction parameters and the characteristic properties of selenium nanoparticles is addressed using a dataset. The dataset was prepared manually by the authors with the aid of data mining from various articles reporting the plant-mediated green synthesis of selenium nanoparticles. The dataset was prepared using Microsoft Excel, and the correlation between reaction parameters and characteristic properties was initially determined using linear regression in Origin 2024b. Mann–Kendall trend test has been employed to identify the increasing or decreasing trend, which is further quantified using Sen's slope estimator. To further verify if correlations truly exist between reaction parameters and characteristic properties, which have been detected using the Mann–Kendall trend test and Sen's slope estimator, an Analysis of Variance (ANOVA) test has been performed. Green-synthesised SeNp is emerging as a significant material for applications in biomedical, electronics, and agricultural fields, which are thoroughly reviewed in this article.

### Plant extracts employed in the green synthesis of SeNp

Different phytochemicals or biomolecules are present in the plant extract, *e.g.*, ascorbic acid, carotenoids, anthocyanins, triterpenoids, and ribosome-inactivating proteins, which can be employed to work as a reducing agent during nanoparticle synthesis.^37^ Hydroxyl and carbonyl groups are present in almost every plant extract, which allows plant extracts to be used as reducing and capping agents.^38^ Besides working as a reducing agent, the phenolic compounds present in the plant extract work as a capping agent in the synthesized SeNp. This capping function enhances the anti-bacterial property, as the phenolic compounds from plant extracts possess anti-bacterial properties, making the resulting SeNp suitable for biomedical applications. In some cases, the plant extracts function as surfactants to stabilise the green-synthesised SeNp.^39^ The most used selenium salts to prepare the precursor solution are sodium selenite, selenious acid, sodium selenate, sodium selenosulphate, selenium sulfide, selenium powder, selenium oxide and selenium sulfate, as reported in the literature. In all cases, the selenium is reduced as selenium nanoparticles upon the reducing action of the plant extract. If the nanoparticles are accurately synthesized, they are dispersed in nature and cannot be seen with the naked eye. The plant extracts commonly employed in the green synthesis of SeNp are illustrated in Fig. 2.

### Green synthesis methodology of SeNp from plant extracts

**Fig. 1:**
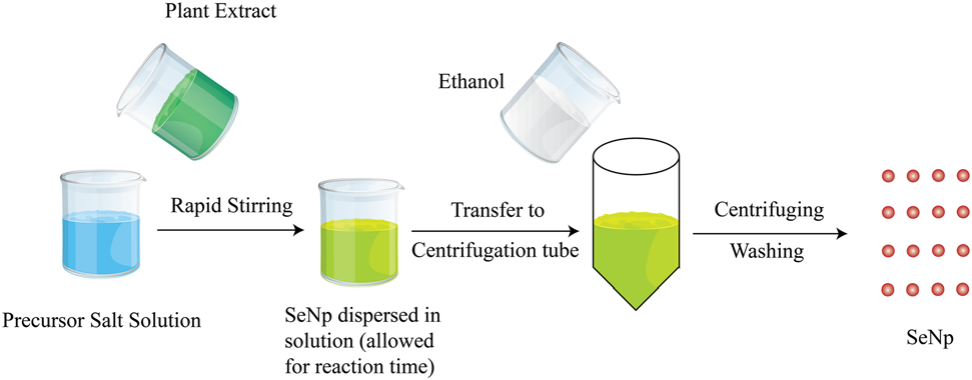
An overview of plant extract mediated green synthesis of SeNp.

The graphical scheme of SeNp production from precursor solution and plant extract is illustrated in Fig. 1.

**Fig. 2:**
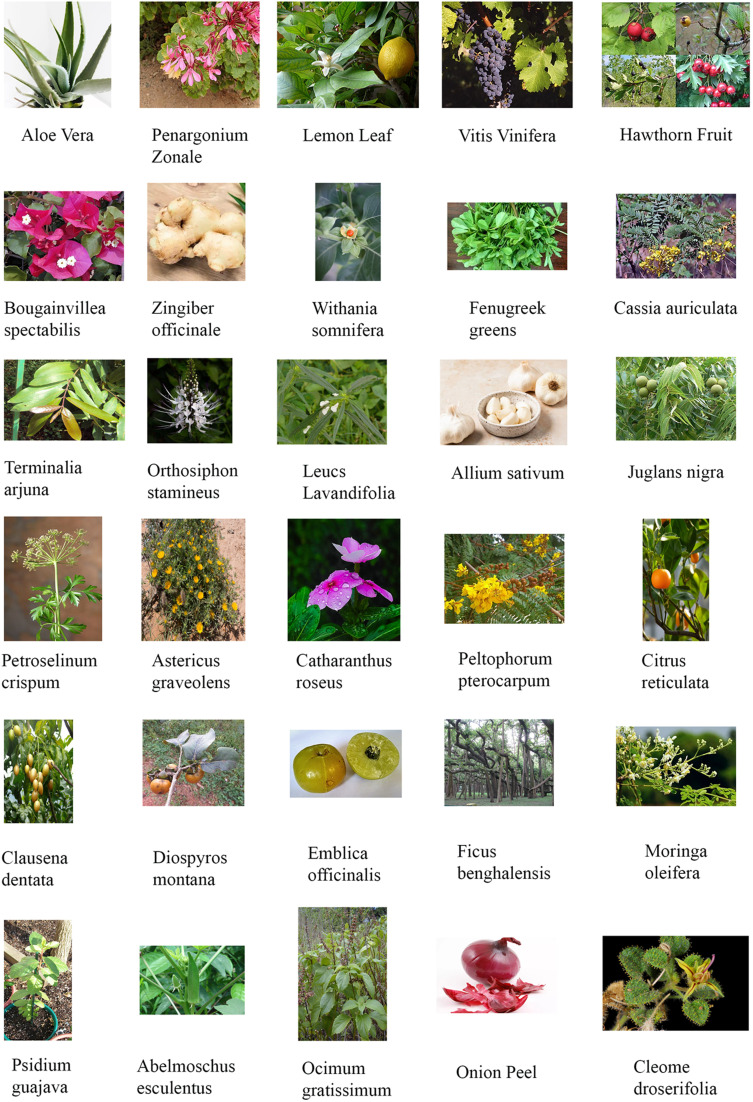
Common plant extracts used in the green synthesis of SeNp.

Plant extracts contain different phytochemical compounds, which work as a reducing agent to reduce the selenium precursor (typically sodium selenite, Na_2_SeO_3_) into zero-valence nano-Se (Se^0^), which cannot be seen with the naked eye. Different functional groups in the plant extracts, such as hydroxyl, carboxyl, and amine groups, aid in the reduction process.^40^ The same phytochemicals also work as capping agents, thus preventing the aggregation of nanoparticles.^41^ The resulting dispersion of SeNp in the extract is then centrifuged to separate the SeNp, or it is passed through a nanofiltration system to separate the SeNp.

### Cost-effectiveness of plant-mediated green synthesis of selenium nanoparticles

Traditional chemical and physical methods are often costly and environmentally harmful due to high energy consumption and the production of toxic waste.^42^ While microbial and algal methods are considered to fall under the domain of green synthesis, they are generally slower and less efficient than plant-mediated green synthesis.^43^ Additionally, the cultivation and maintenance of microbial cultures can be costly.^44^ Compared to the mentioned processes, plant-mediated-green synthesis is more cost-effective because plant extracts are readily available and inexpensive.^45^ Additionally, the plant-mediated green synthesis process is straightforward, which requires only basic laboratory equipment and reduces cost.^46^ Plant-mediated synthesis is generally faster than microbial and algal methods,^47^ and the capping action of phytochemicals present in plant extracts acts as a natural stabilizer of the resulting nanoparticles. Considering all these factors, the plant-mediated green synthesis process of selenium nanoparticles can be termed the most cost-effective and has been selected for analysis in this study.

### Important characteristic parameters of SeNp synthesis

The unique attributes of nanoparticles in different applications are highly dependent on the characteristic parameters that define their end use, such as size, shape, and surface properties.^48,49^ In this article, we will focus on the core size, hydrodynamic diameter, zeta-potential, and polydispersity index as the characteristic properties of SeNp. Hydrodynamic diameter is the diameter of the nanoparticles in water (or solvent), determined by dynamic light scattering (DLS).^50^ By DLS, we do not get the core size of the nanoparticle but the overall diameter, including layers of adsorbed water. By transmission electron microscopy (TEM),^51^ or scanning electron microscopy (SEM) (when particle size > 50 nm),^52^ or atomic force microscopy (AFM),^52^ we get the core size of the nanoparticles. Zeta-potential is defined as the potential difference between the stern layer and diffuse layer of a particle in solution.^53^ Zeta-potential is measured to find the charge of the nanoparticles, and it is a significant property because if the zeta-potential value is more negative or more positive, then there would be electrostatic repulsion and thus, the nanoparticles will not form aggregates.^54^

The size of nanoparticles is significant, as it defines their availability and corresponding application.^55^ The size distribution of the synthesized nanoparticles is explained by the polydispersity index (PDI), whereby a PDI value in the range of 0.1 to 0.25 indicates a narrow size distribution. In contrast, PDI> 0.5 indicates a broad particle size distribution.^56^ The polydispersity index is a measure of how dispersed the nanoparticle sizes are, and if there is uniform size distribution, the PDI value would be close to 0.1. The morphology describes the shape of the selenium nanoparticles, which is spherical primarily, as reported in the literature. However, nanoballs,^57,58^ oval,^41^ rod,^59^ hexagonal,^60^ and flower shaped particles are also found in the literature.^37^ The crystallinity index serves as an indication of the degree of crystallinity, or how much the nanoparticles have crystallized and is equal to the ratio of the area under the crystalline peak and the area under all peaks in the XRD peak pattern. However, to the best of our knowledge, the crystallinity index of green synthesized SeNp was reported in only one article, which was 66.80%.^54^ The rest of the articles provided the XRD peak pattern, some of which included crystallite size from the Debye–Scherrer equation. However, due to the greater degree of amorphous nature of the green synthesized SeNp (perhaps due to the capping action of the plant extract), the crystallinity index was not determined. The remaining characteristics described are important for the subsequent application of selenium nanoparticles, and they are highly dependent on the operational parameters.

### Statistical analysis using Mann–Kendall trend test with Sen's slope estimator and analysis of variance (ANOVA)

The Mann–Kendall (MK) trend test is a proper statistical analysis tool used to detect any monotonic upward or downward trend.^61^ It has been reported to be used for detecting an increase or decrease in temperature,^62^ rainfall,^63^ and other hydrologic data with the increase of time.^64^ Fireworks are a source of nanoparticles, particulate matter, and carbonaceous aerosols, and the trend between the concentration of particulate matter with aerodynamic diameter up to 10 μm and time has been reported in the literature, where Mann–Kendall trend test was performed to observe any trend.^65^ There are two types of hypotheses in the Mann–Kendall test: one is a null hypothesis, and the other is an alternate hypothesis. The null hypothesis implies there is no trend in the dataset, whereas the alternate hypothesis implies there is an increasing or decreasing trend in the dataset.^66^ In most cases, a significance value of 0.05 is used, which implies there is less than a 5% chance of a null hypothesis existing in the dataset.^67^ If the resulting *p*-value (two-tailed) is less than 0.05, then the alternate hypothesis is accepted (null hypothesis is rejected). The existence of a linear upward or downward trend is accepted, and if it is greater than 0.05, then enough evidence is not there to reject the null hypothesis. Thus, there is no monotonic upward or downward trend in the dataset.^68^ The Kendall's tau (*τ*) and test statistic (*S*) parameters indicate the direction and strength of the trend in the series, whereby a positive sign indicates an increasing trend, a negative sign a decreasing trend, and the higher value of *τ* and *S*, the greater strength of the trend.^69^ To determine Mann–Kendall's tau (*τ*), for each pair of observations (*i*, *j*), where *i* < *j*, it is first determined if the pair is concordant or discordant. A pair is termed concordant if the ranks of both observations increase together and discordant if one rank increases while the other decreases. The number of concordant pairs and discordant pairs are then counted. Then, using the following formula, Kendall's tau (*τ*) is calculated:
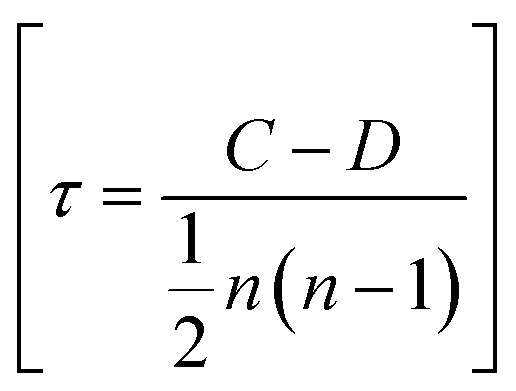
where (*n*) is the number of observations. For calculating the test statistic (*S*), for each pair of data points (*x*_*i*_, *x*_*j*_) where *i* < *j*, the sign function is computed using the following cases:
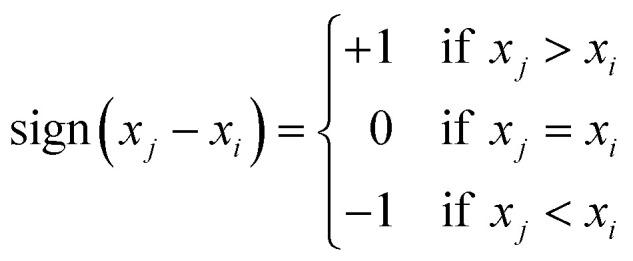


Then, the results of the sign function over all pairs are summed to get the test statistic (*S*) using the following formula:
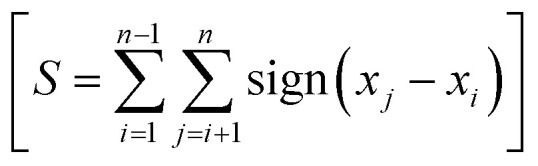


The parameter Var(*S*) is implied for determining the variance of *S*.^70^ If there are no ties (equal values) in the dataset, then the parameter Var(*S*) is calculated using the following formula:
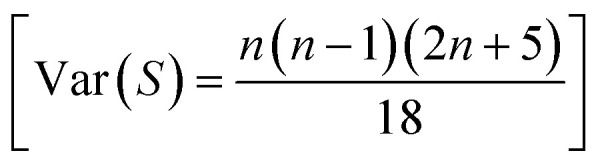
If there are ties or equal values, the variance is adjusted to account for them by the following formula:
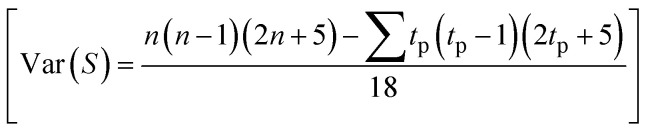
Here, *t*_p_ is the number of ties of extent *p*. In this article, the Mann–Kendall (MK) test is performed to assess the causality between input and output parameters. This test provides a more reliable assurance of causality between the input and output parameters after the results from conventional graph plotting have been assessed. The input parameters like temperature, stirring speed, reaction time, and incubation period are given entry in the data field, and the output parameters core size, hydrodynamic diameter, zeta potential, and polydispersity index were given entry in the “Time series” field. The significance value (α) of 0.05 is used, and the correlation between the input and output parameters is thus assessed. Sen's slope is used to quantify the trend observed using the Mann–Kendall trend test, which depicts the change in the output parameter with a unit change in the input parameter.^69^ For each pair of data points, (*x*_*i*_, *y*_*i*_) and (*x*_*j*_, *y*_*j*_), the slope *S*_*ij*_ is calculated using the following formula:
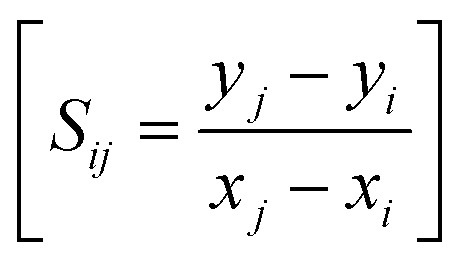


The Sen's slope estimator takes the median of all the pairwise slope *S*_*ij*_. The lower and upper bound in Sen's slope estimator represents a 95% confidence interval for the slope coefficient, which corresponds that there is 95% assurance that the actual coefficient falls between the lower and upper bounds, and the data could be interpreted accordingly.^71^ The magnitude of Sen's slope can also indicate the strength of the trend, and the sign would depict the positive or negative trend in the parameters.^69^ Sen's slope is considered better for detecting linear relationships because it is not affected by outliers in the data.^72^ Noted that the Mann–Kendall (MK) trend test does not necessarily confirm the causality between the input and output parameters but increases the chance of one existing.

Analysis of variance (ANOVA) test is a statistical method used to compare the means of three or more groups to identify if there are any statistically significant differences among them.^73^ There are multiple types of ANOVA. The one-way ANOVA tests the effect of a single independent variable on each dependent variable(s) and is used when the dependent variable(s) relies on one factor.^74^ Two-way ANOVA is used when there are two independent variables which affect the dependent variable.^75^ Multi-way ANOVA extends the two-way ANOVA to more than two factors or independent variables and is used when more than two independent variables are affecting the dependent variable(s),^76^ and multivariate ANOVA (MANOVA) is used when the effect of two or more independent variables on two or more dependent variables are determined simultaneously, *e.g.*, in this case, it is assumed that the dependent variables also rely on each other.^77^ The *F*-statistic is a key parameter to interpret the ANOVA results, which represents the ratio of variance between groups to the ratio of variance within groups. If the *F*-statistic value is larger than the critical value, then the null hypothesis is rejected, which represents that there is a strong correlation.^78^ In the case of two-way ANOVA, the interaction effects are considered. Suppose the *F*-statistics, partial eta-squared (*η*^2^) or omega squared (*ω*^2^) value is larger than the main effects considered in one-way ANOVA. In that case, the interaction is considered to have a more substantial impact on the dependent variables.

### Data mining and curation

The reaction parameters and characteristic properties analyzed in this study are extracted manually from research articles in the literature. No help of any software is taken during data extraction because there is a chance of error in the extracted data. Only articles related to the plant-extract-mediated green synthesis of selenium nanoparticles are chosen for data extraction, as this is the topic of our study. Next, the dataset is curated to retrieve data where reaction parameters exhibit a strong correlation with characteristic properties, and these data are thoroughly analyzed. The tables containing data are available in SI Document.

### Influence of volume and concentration (plant extract and precursor solution) on particle size, zeta potential and polydispersity index (PDI)

The volume and concentration of both plant extract and precursor solution have a significant impact on the characteristic properties of SeNp. The concentration of plant extract is directly related to the size of the synthesized nanoparticles, and researchers have found that increasing plant extract concentration will decrease the size of the nanoparticles because a greater number of biomolecules are available that will cap the nanoparticles and prevent them from forming agglomerates.^79^ However, as the nucleation process is faster than particle development at greater concentrations if a more potent reducing agent is employed, a smaller SeNp size is expected to be obtained.^80^ But no significant trends can be observed for different plant extracts because they contain varying degrees of phytochemicals. To the best of our knowledge, green synthesized SeNp from aloe vera leaf extract (ALE),^81^ and *Pelargonium zonale* leaf extract was reported in the literature,^82^ where the concentration of the precursor solution and plant extract was kept fixed, while the volume of both the precursor solution was varied to find the optimum volume. The data is summarized in SI Document (Table S1).

**Fig. 3:**
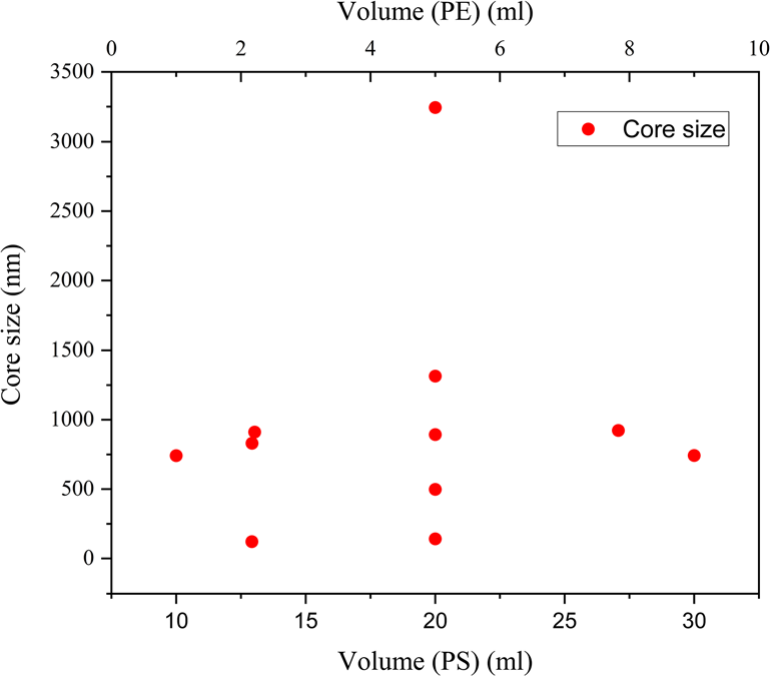
Dependence of volume (mL) on core size (nm) of SeNp synthesized from aloe vera leaf extract.

**Fig. 4:**
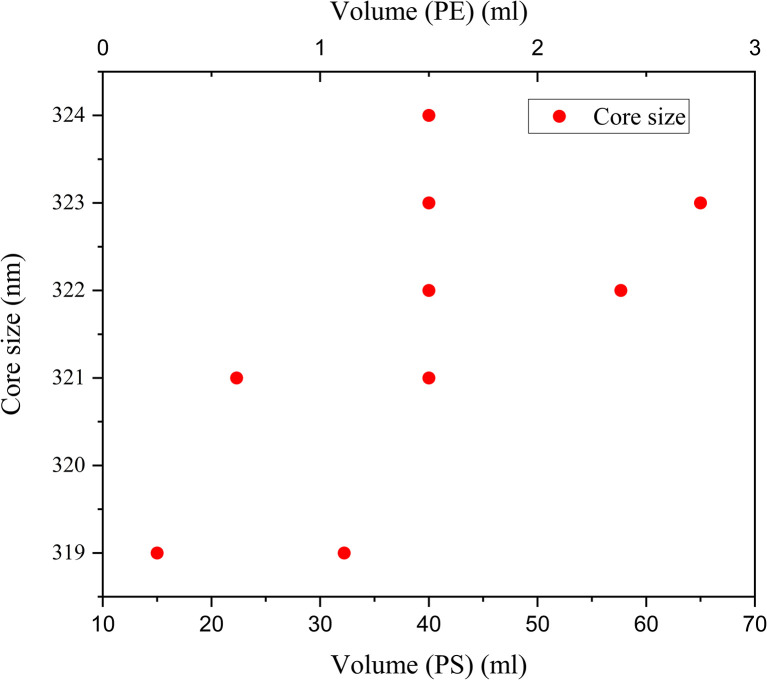
Dependence of volume (mL) on core size (nm) of SeNp synthesized from *Pelargonium zonale* leaf extract.

The volume was varied in both the cases of Aloe-vera leaf extract and *Pelargonium zonale* leaf extract and no significant trend could be observed; instead, the volumes were optimized to get the smallest SeNp core size, of which the zeta-potential and polydispersity index was determined. The influence of volume on zeta potential and polydispersity index could not be analysed due to lack of data, and influence on particle size in the case of a fixed plant extract was reported in another study,^81^ both of which rendered irregular results, which are plotted in Fig. 3 and 4.

In one study, an interesting case was observed in the case of green-synthesised SeNp from chamomile extract, mint extract, green tea extract, and black tea extract.^83^ In the case of chamomile extract and mint extract, concentration was not defined; the volumes were shuffled by first fixing the precursor solution volume as 2.5 mL and varying the plant extract volumes 2.5, 5, 7.5 and 10 mL and then fixing the plant extract volume as 2.5 mL and varying the precursor solution volume in the order 5, 7.5 and 10 mL. In the case of chamomile extract, while varying the plant extract volume, the core SeNp size observed was 95.4 nm, and a hydrodynamic diameter of 120 nm. When the plant extract volume was kept constant at 2.5, and the precursor solution volume increased, the hydrodynamic diameter increased to 125.9 ± 5.31 nm. An opposite phenomenon was observed in the case of mint extract, whereby increasing plant extract volume increased hydrodynamic diameter, whereas increasing the volume of precursor solution resulted in a decrease in hydrodynamic diameter. The same pattern of volume was followed in the cases of black tea and green tea extract, whereby concentration was also mentioned. The same pattern of volume was followed in the cases of black tea and green tea extract, whereby concentration was also mentioned. It was followed that increasing both the concentration and volume of the plant extract increased the hydrodynamic diameter of the resulting SeNp, whereas increasing the precursor solution volume resulted in a subsequent decrease in the hydrodynamic diameter. In all the cases stated, 100 mM Na_2_SeO_3_ solution was used as the precursor solution; the polydispersity index was also addressed in synthesis of SeNp from the four extracts mentioned but did not follow any increasing or decreasing pattern.^83^

Several strategies can be employed to determine the specific trend of characteristic properties related to the volume and concentration of plant extract and precursor solution, which opens up a scope for future studies. Standardized phytochemical profiling may be performed, in which quantitative analysis of key reducing and capping agents in plant extracts using the HPLC-MS technique will allow the determination of a correlation between specific phytochemical concentrations rather than total extract volume and the resulting characteristic properties of SeNp.^84^ Response surface methodology (RSM) and design of experiments (DoE) approaches may be employed to simultaneously evaluate the interaction between precursor concentration, plant extract volume, concentration, and synthesis conditions, rather than a one-way approach.^85^*In situ* characterization techniques, such as UV-vis spectroscopy, dynamic light scattering (DLS), and electrochemical monitoring during synthesis to track nucleation kinetics and growth mechanisms, which can help better understand the volume and concentration-dependent effects.^86^ Surface-sensitive techniques, such as FTIR, XPS, and structural analysis, *e.g.*, SAXS/WAXS combined with computational modeling and machine learning strategies, can potentially identify biomolecule-nanoparticle binding mechanisms and develop predictive models for synthesis optimization.^87^

### Influence of temperature

#### Influence on particle size

As stated previously in the literature,^1^ with an increase in temperature, a decrease in the resulting nanoparticles is usually observed. In the case of selenium nanoparticles, based on the previous literature data, although the decreasing nanoparticle size with increasing temperature trend is not linear but scattered due to different precursor solution and plant extract type, a sequence can still be observed, which is summarized in SI Document (Table S2).

**Fig. 5:**
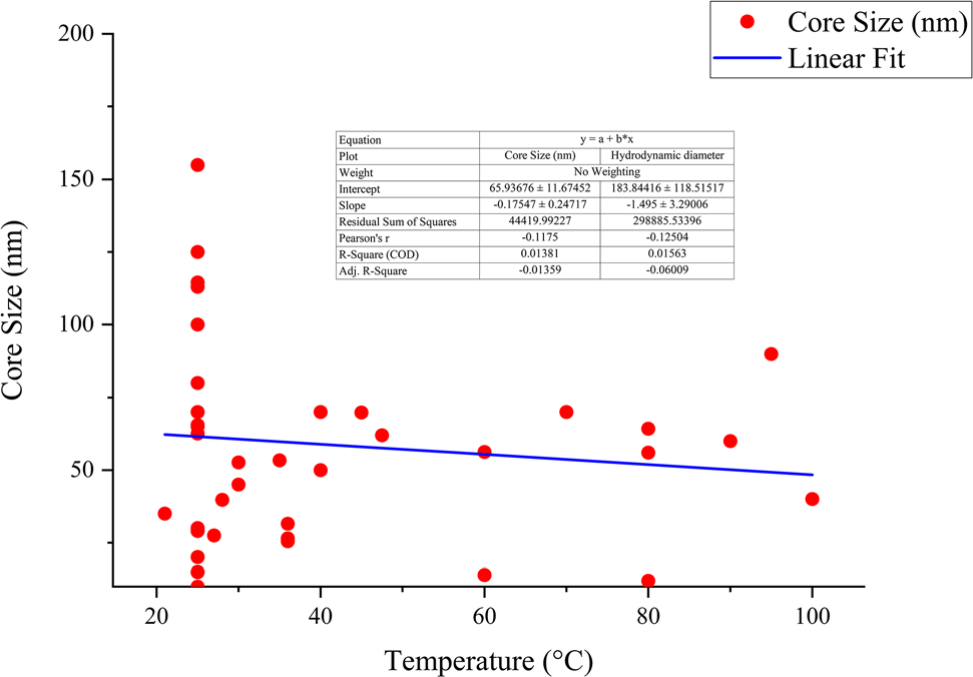
Correlation between temperature (°C) and core size (nm).

**Fig. 6:**
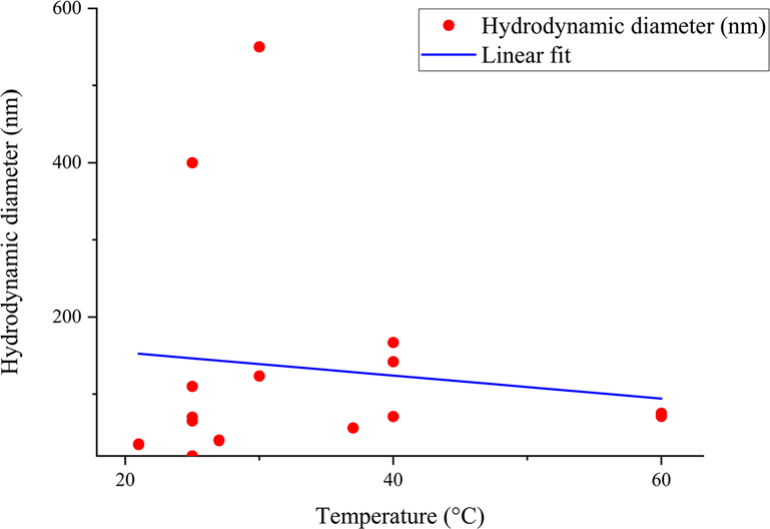
Correlation between temperature (°C) and hydrodynamic diameter (nm).

The correlation between reaction condition and core size (nm) along with hydrodynamic diameter (nm) is represented in Fig. 5 and 6. From the figure plotted, it was clear that due to the interrelated nature of the different operational parameters, an abruptly scattered nature was observed in their correlation. Based on the reviewed data in SI Document (Table S2), the authors suggest that the lowest recommended temperature range for green synthesized SeNp be 20–22 °C, which is recommended in the literature where Roselle plant leaf extract was used, and a core size of 35 nm and hydrodynamic diameter in the range of 20–50 nm has been reported.^88^ If the temperature is decreased further, it can slow down the reaction kinetics, leading to increased aggregation and larger nanoparticle size.^89^ Additionally, based on the reviewed data, the authors suggest that the optimum temperature for plant-mediated green synthesis of SeNp might be in the range of 60–70 °C. Suppose the temperature is increased to a great extent. In that case, some phytochemical compounds in the plant extract might break down (as they are organic compounds that decompose at elevated temperatures), resulting in the loss of reduction capacity and capping ability of the phytochemical compounds.

**Fig. 7:**
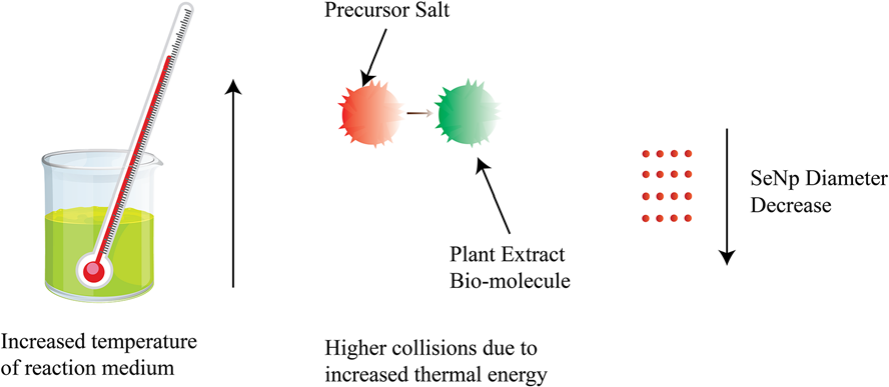
Decrease of SeNp diameter with the increase in temperature.

The relationship between temperature and nanoparticle size (diameter) is schematically represented in Fig. 7.

In one study, selenium nanoparticles synthesized under microwave heating have been reported to exhibit higher antioxidant activity (44.2%) compared to those synthesized using conventional methods (34.8%) and an autoclave method (32.6%).^90^ The results indicate that an increase in synthesis temperature improves the quality of selenium nanoparticles synthesized for biomedical applications.

#### Influence on zeta-potential

The zeta potential indicates the stability of the resulting nanoparticles. It is a measure of electric charge on the nanoparticle's surface and helps quantify the charges.^91^ The zeta potential of green synthesized SeNp at different temperatures is summarized in SI Document (Table S3).

Zeta potential is the potential difference between the stern layer and the diffuse layer of a particle. When the selenium nanoparticle is reduced from the precursor solution, the net charge of SeNp is zero. However, the reducing agents, that is, the plant extract, also work as a capping agent, making the SeNp surface either positively or negatively charged, depending on the phytochemicals present. The zeta potential depends on the fluid in which the SeNp is dissolved. If the reaction medium in which the reaction is taking place is at a relatively high temperature, then due to enhanced diffusion of phytochemicals and activation of the functional groups, the surface charge of the SeNp is expected to increase either too positive or too negative, which indicates the stability of the SeNp in a solution and no tendency to form aggregates.^53^ When the SeNp is separated from the reaction medium and zeta-potential assessed by dissolving in ideal solvent water, then H^+^ from the water forms a stern layer, which is a compact layer, and OH^−^ ions and H^+^ ions in the diffuse layer are weakly bounded and free to move. Thus, the more negatively charged or positively charged the SeNp surface is, the more counteracting ion in the stern layer, and thus more potential difference from the diffuse layer; that is, zeta potential is expected to increase with the increase in temperature of the reaction medium.

**Fig. 8:**
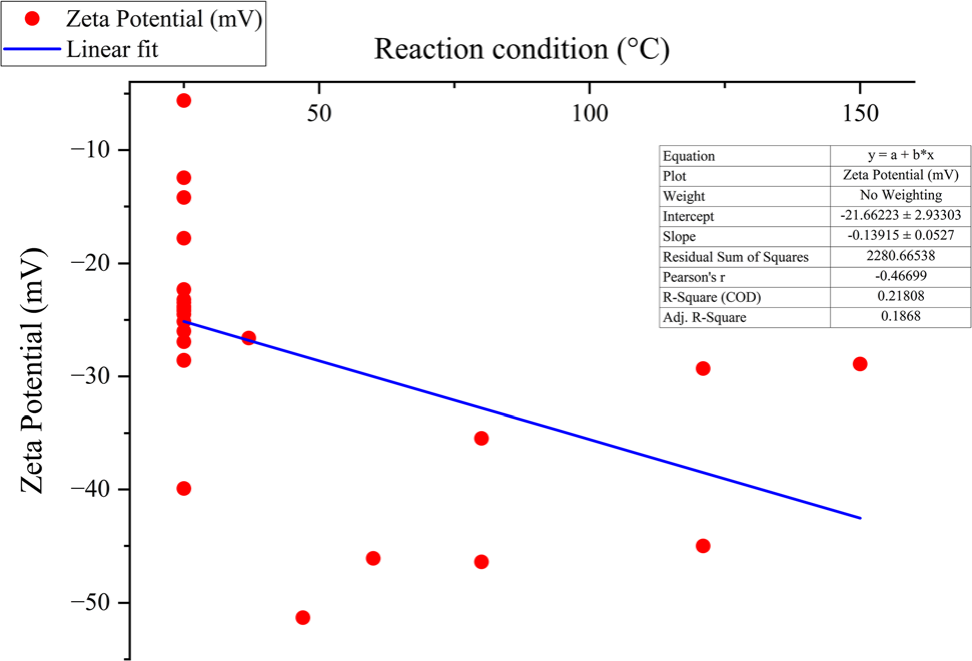
Correlation between zeta potential (mV) and temperature (°C).

However, it mainly depends on the phytochemicals present in the plant extract, and thus, a linear trend is not expected; instead, a non-linear behaviour of more positive or more negative zeta potential with increased temperature is expected to occur as we assess green-synthesized SeNp from different plant extracts. This is plotted in Fig. 8.

**Fig. 9:**
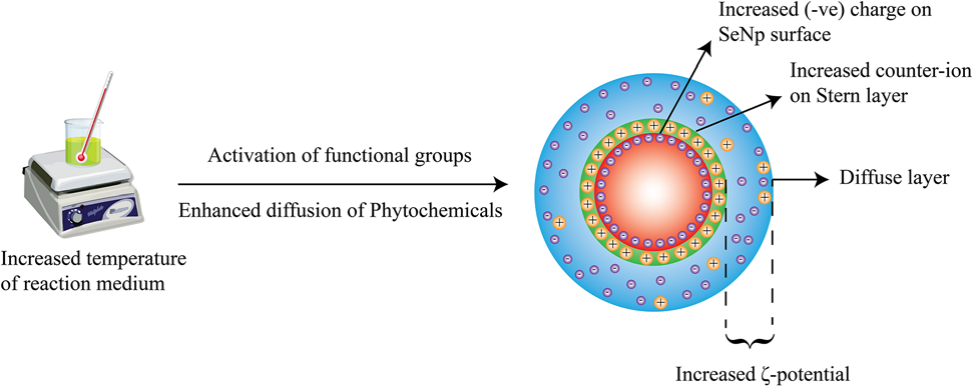
Increasing zeta potential (more positive or more negative) upon increasing the temperature of reaction medium.

The overall phenomenon ascribed is schematically summarized in Fig. 9.

#### Influence on polydispersity index (PDI)

The polydispersity index represents the homogeneous distribution of nanoparticle size. When the size distribution is almost uniform, the polydispersity index value approaches 0. Highly polydisperse samples have a polydispersity index (PDI) close to 1. The PDI, or polydispersity index is defined by the formula *μ*^2^/*Γ*^2^, where *μ* is the deviation and *Γ* is the mean size. This equation indicates the normalized width of the Gaussian distribution.^92^ The effect of temperature on the PDI value of green synthesized SeNp is collected from different articles in the literature and summarized in SI Document (Table S4).

**Fig. 10:**
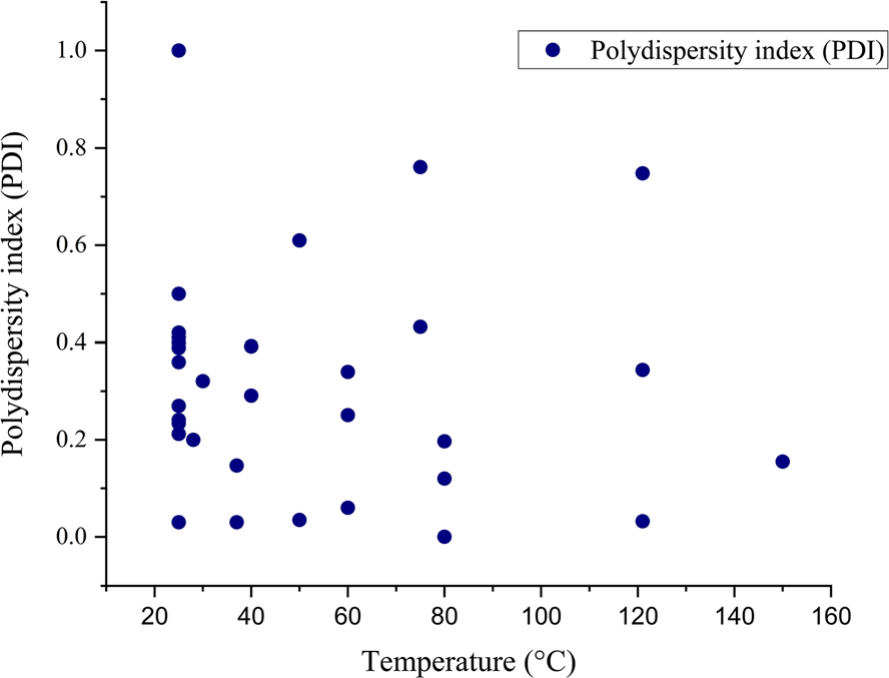
Scattered data plotting of polydispersity index (PDI) *vs.* temperature (°C).

The type of the plant extract and phytochemicals present, concentration of the precursor solution and plant extract, and the stirring speed do affect this parameter. This phenomenon is plotted in Fig. 10.

#### Mann–Kendall trend test for influence of temperature

The Mann–Kendall trend test for temperature and core size implied a *p*-value (two-tailed) of 0.039, temperature and hydrodynamic diameter yielded a *p*-value (two-tailed) of 0.721, temperature and zeta-potential rendered a *p*-value (two-tailed) of 0.000, and temperature and polydispersity index rendered *p*-value (two-tailed) of 0.213. The *τ* and *S* values for temperature and core size are −0.376 and −51, respectively. Combined with the *p*-value for temperature and core size, these values coincide with the observation found from conventional graph plotting, which is a strong decreasing trend of core size with increasing temperature. In contrast, the *τ* and *S* values for temperature and hydrodynamic diameter are 0.111 and 5, respectively. However, the *p*-value of temperature and hydrodynamic diameter proposes an opposite phenomenon (null hypothesis, no trend) from that of conventional graph plotting (trend observed). The *τ* and *S* values for temperature and zeta potential are −0.539 and −136, respectively, and combined with the *p*-value (two-tailed) for temperature and zeta-potential, coincides with the observation obtained from conventional graph plotting and confirms the presence of an increasing trend (more negative) zeta potential with the increase in temperature. The *τ* and *S* values for temperature and polydispersity index are −0.152 and −85, respectively. However, the *p*-value for temperature and polydispersity index suggests the presence of no trend (null hypothesis). The conventional graph plot also renders scattered data for the correlation between temperature and polydispersity index. Thus, the result of the Mann–Kendall trend test confirms that there is no trend or correlation between temperature and the polydispersity index. Comparing the *τ* and *S* values for temperature and core size and zeta potential, we can assume that zeta-potential shows a more abrupt increase (more negative) trend with temperature than the core size decreases upon the increase of temperature. The reason for the null hypothesis observation of hydrodynamic diameter (nm), which is contradictory to what is observed from conventional graph plotting, is perhaps the lack of adequate data. The Mann–Kendall trend test data for temperature (°C) and core size (nm), hydrodynamic diameter (nm), zeta-potential (mV) and polydispersity index PDI is summarized in Table 1.

**Table 1:** Mann–Kendall trend test data for correlation between temperature (°C) and core size (nm), hydrodynamic diameter (nm), zeta-potential (mV) and polydispersity index (PDI)

Parameter	Core size (nm)	Hydrodynamic diameter (nm)	Zeta potential (mV)	Polydispersity index (PDI)
Kendall's tau	−0.376	0.111	−0.539	−0.152
*S*	−51	5	−136	−85
Var (*S*)	588.333	125.000	1432.667	4548.333
*p*-Value (two-tailed)	0.039	0.721	0.000	0.213
*α*	0.05	0.05	0.05	0.05

#### Sen's slope analysis for influence of temperature

From the Mann–Kendall trend test, it has been observed that the core size (nm) and zeta potential (mV) exhibit a significant decreasing trend with increasing temperature. The decreasing trend is further confirmed using Sen's slope estimator, where the core size (nm) *vs.* temperature (°C) graph yielded a Sen's slope of −1, whereby zeta-potential (mV) *vs.* temperature (°C) graph yielded a Sen's slope of −0.205. The sign of Sen's slope confirms the decreasing trend. In contrast, core size (nm) shows a more abrupt decreasing trend according to Sen's slope estimation, which is a more reliable quantification of increasing/decreasing trend. Overall, the Sen's slope test data is summarized in Table 2.

**Table 2:** Sen's slope test data for correlation between temperature (°C) and core size (nm), hydrodynamic diameter (nm), zeta-potential (mV) and polydispersity index (PDI)

Parameter	Core size (nm)	Hydrodynamic diameter (nm)	Zeta potential (mV)	Polydispersity index (PDI)
Sen's slope	−1	0.006	−0.205	−0.002
Lower bound (95%)	−1.990	−2.613	−0.073	−0.002
Upper bound (95%)	0.232	4.970	0.000	0.004

**Fig. 11:**
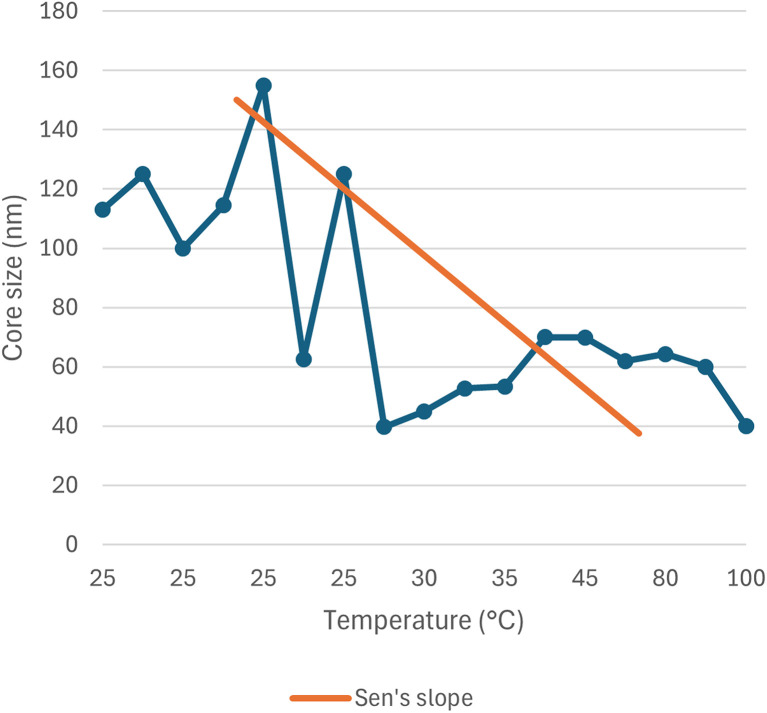
Correlation between temperature (°C) and core size (nm) using Mann–Kendall trend test.

**Fig. 12:**
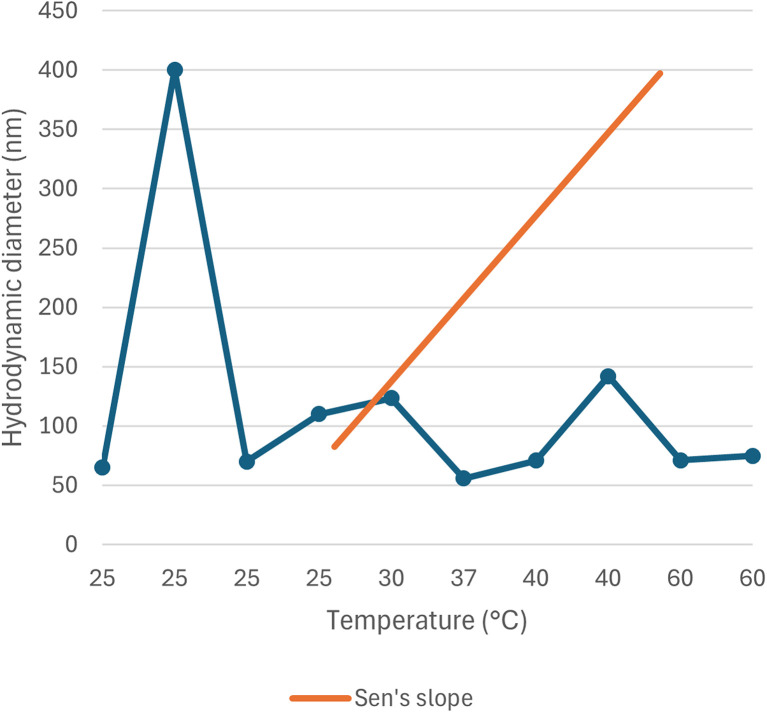
Correlation between temperature (°C) and hydrodynamic diameter (nm) using Mann–Kendall trend test.

**Fig. 13:**
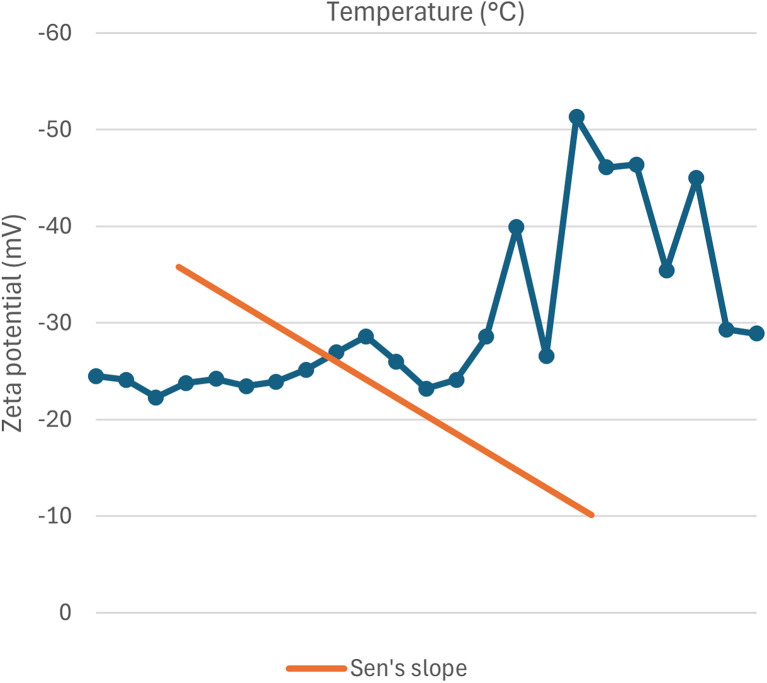
Correlation between temperature (°C) and zeta-potential (mV) using Mann–Kendall trend test.

**Fig. 14:**
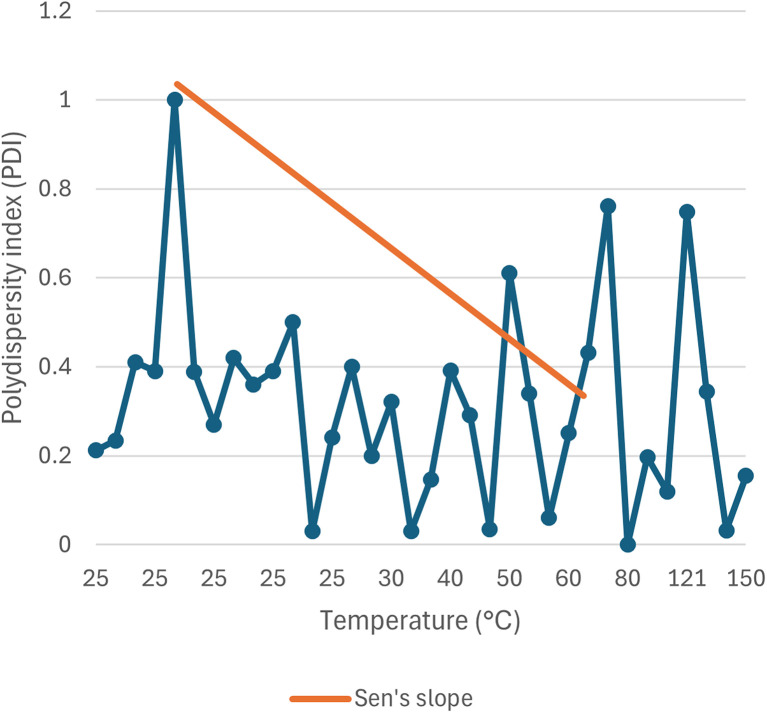
Correlation between temperature (°C) and polydispersity index (PDI) using Mann–Kendall trend test.

The correlation between temperature (°C) and core size (nm), hydrodynamic diameter (nm), zeta potential (mV) and polydispersity index (PDI) is plotted in Fig. 11–14.

#### ANOVA for temperature

By performing a one-way ANOVA between temperature (independent variable) and core size (dependent variable), an *F*-statistic value of 2.426 and a Pr(>*F*) value of 0.128 have been obtained. Thus, we fail to reject the null hypothesis, as the Pr(>*F*) value is greater than the significance value *α* (0.05). Thus, the temperature and core size relation is not statistically significant according to the ANOVA test.

One-way ANOVA between temperature and zeta potential rendered an *F*-statistic value of 7.949 and a Pr(>*F*) value of 0.000431, which indicates the relation between temperature and zeta potential is statistically significant, and we can reject the null hypotheses.

### Influence of stirring speed

#### Influence on particle size

With the increase in stirring speed, the nanoparticles synthesised are expected to show a decreasing trend in size because rapid stirring would instantaneously churn the nanoparticles formed and prevent any agglomeration from occurring. This theorem was first proposed in earlier works on the synthesis of nanoparticles,^93^ and a similar trend was observed in the green synthesis of selenium nanoparticles, which is summarised in SI Document (Table S5).

**Fig. 15:**
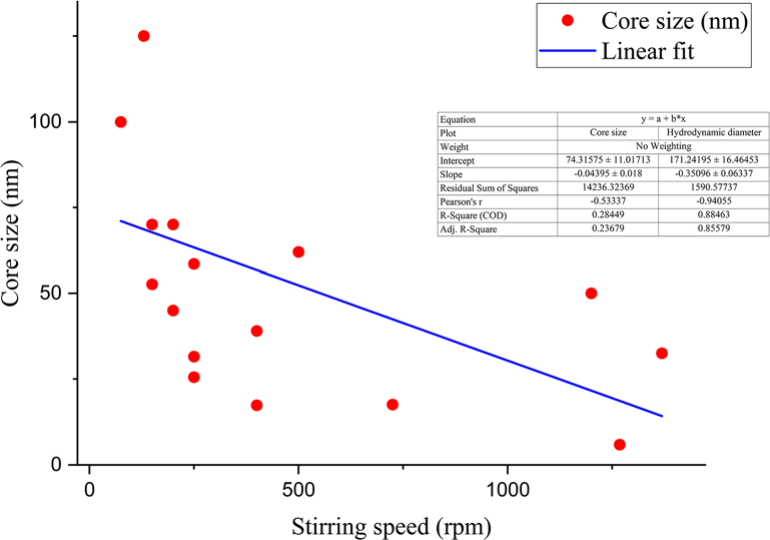
Correlation between stirring speed (rpm) and core size (nm).

**Fig. 16:**
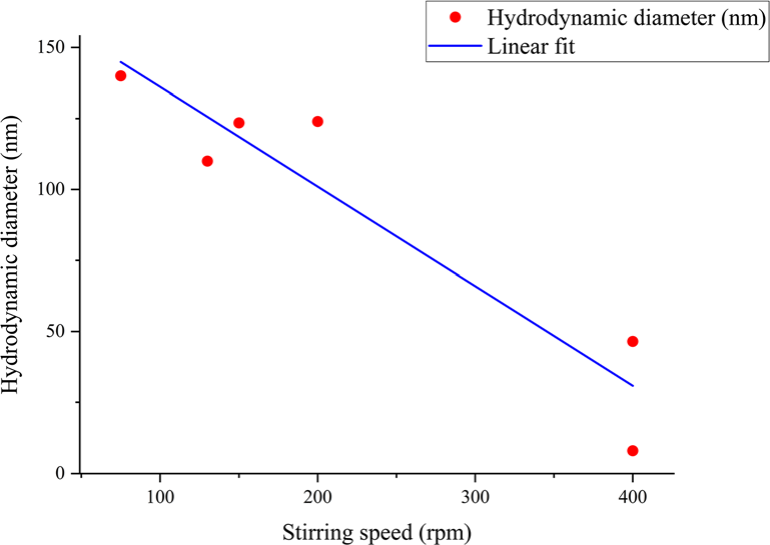
Correlation between stirring speed (rpm) and hydrodynamic diameter (nm).

Although the trend of decreasing particle size with an increase in stirring speed is not linear due to the influence of the reducing agent (capping agent as well), concentration of the precursor solution, reaction time, *etc.*, as they are interdependent variables, a non-linear trend of decreasing particle size with increasing stirring speed is observed from the data of the previous literature, which is schematically plotted in Fig. 15 and 16.

**Fig. 17:**
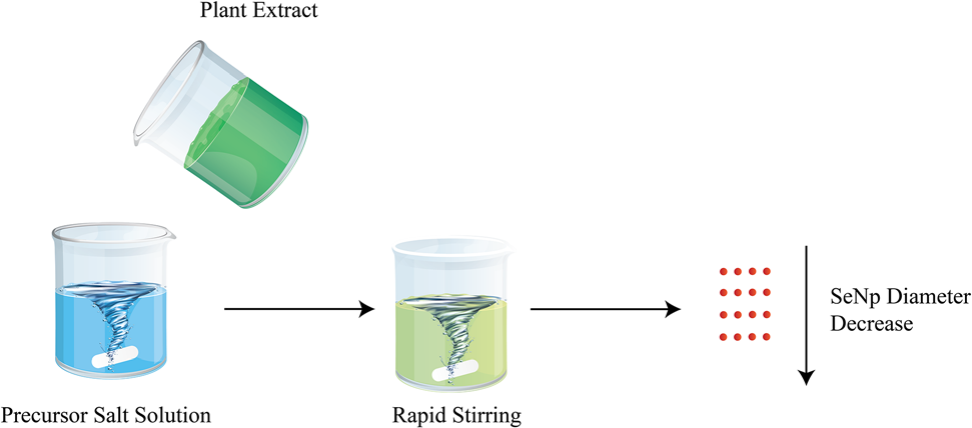
Decrease in diameter of green synthesized SeNp with increased stirring speed.

The overall phenomenon is schematically presented in Fig. 17.

#### Influence on zeta-potential

Usually, increasing the stirring speed should increase the zeta-potential value because the contact between the capping agent (phytochemicals) and synthesized SeNp becomes more abrupt, resulting in a higher surface charge density.^94^ The relationship between stirring rate and zeta potential from previous data in the literature is summarized in SI Document (Table S6).

**Fig. 18:**
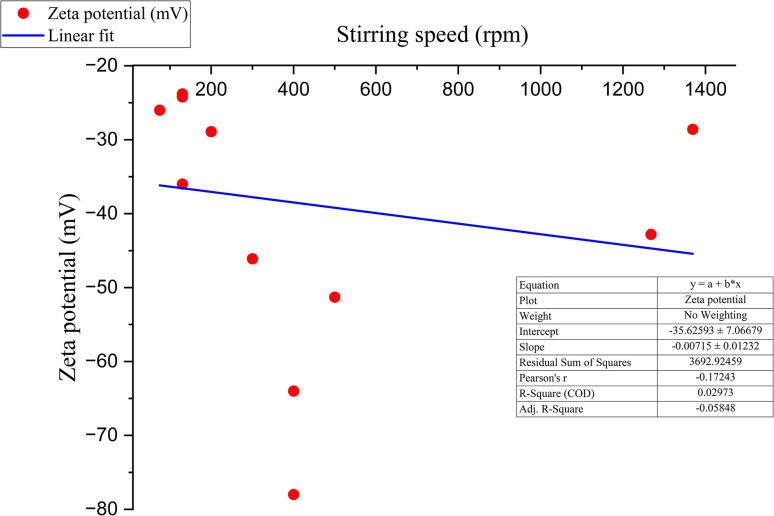
Correlation between zeta potential and stirring speed of green synthesized SeNp.

Although not exactly linear, a scattered yet significant relationship is observed in this case, where the increase in stirring speed results in a more negative zeta potential value. This correlation between zeta potential and stirring speed is plotted in Fig. 18.

**Fig. 19:**
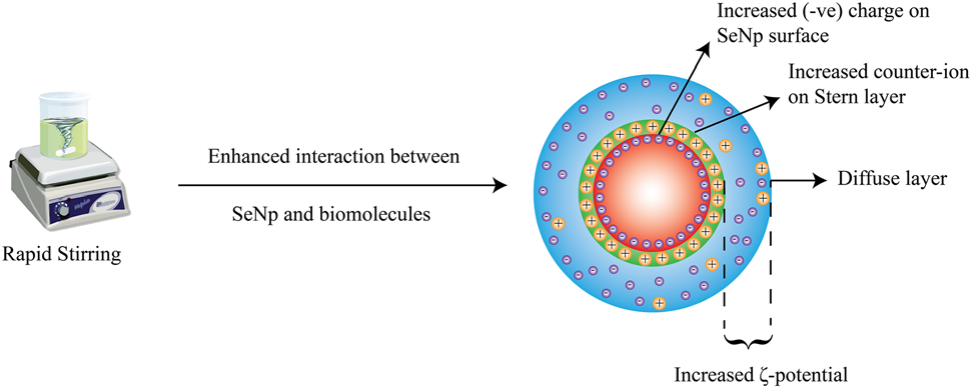
Increased zeta-potential of SeNp upon the influence of rapid stirring action.

This overall phenomenon is schematically presented in Fig. 19.

#### Influence on polydispersity index (PDI)

Theoretically, upon the action of rapid stirring, the size distribution of SeNp is expected to be 0.1; that is, the particles are expected to be monodisperse, as the action of rapid stirring churns away the formation of any agglomerates. The stirring speed and corresponding PDI value for green-synthesised SeNp, as collected from the literature, are summarised in SI Document (Table S7).

**Fig. 20:**
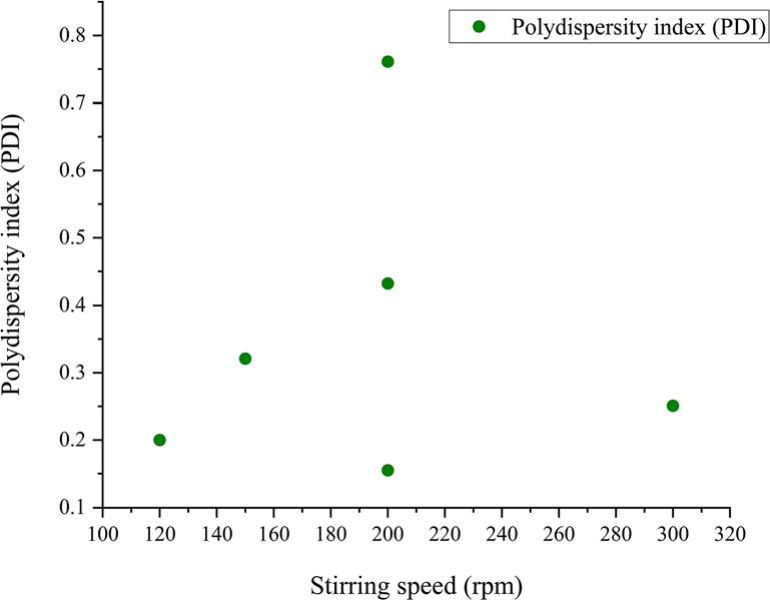
Scattered relationship between polydispersity index (PDI) and stirring speed of green synthesized SeNp from plant extracts.

Inadequate data are available regarding the correlation between stirring speed and polydispersity index (PDI) for green-synthesised SeNp, and the existing data also exhibit extremely non-linear behaviour. Thus, although theoretically, the PDI value is expected to decrease with an increase in stirring speed, no conclusion can be drawn for the case of green-synthesised SeNp. The relationship between the polydispersity index (PDI) and stirring speed is plotted in Fig. 20.

#### Mann–Kendall trend test for influence of stirring speed

The Mann–Kendall trend test for stirring speed and core size implied a *p*-value (two-tailed) of 0.002, stirring speed and hydrodynamic diameter yielded a *p*-value (two-tailed) of 0.133, stirring speed and zeta-potential rendered a *p*-value (two-tailed) of 0.019, and stirring speed and polydispersity index rendered *p*-value (two-tailed) of 1. The *τ* and *S* values for stirring speed and core size are −0.563 and −76, respectively. Combined with the *p*-value for stirring speed and core size, these results coincide with the observation found from conventional graph plotting, which shows a strong decreasing trend of core size with increasing stirring speed. In contrast, *τ* and *S* values for stirring speed and hydrodynamic diameter are −0.600 and −9, respectively. However, the *p*-value of stirring speed and hydrodynamic diameter proposes an opposite phenomenon (null hypothesis, no trend) from that of conventional graph plotting (trend observed). The *τ* and *S* values for stirring speed and zeta-potential are −0.534 and −35, respectively, and combined with the *p*-value (two-tailed) for stirring speed and zeta-potential, coincides with the observation obtained from conventional graph plotting and confirms the presence of a strong increasing trend (more negative) zeta potential with the increase in stirring speed. The *τ* and *S* values for stirring speed and polydispersity index are 0.067 and 1, respectively. However, the *p*-value for stirring speed and polydispersity index suggests the presence of no trend (null hypothesis). The conventional graph plot also renders scattered data for the correlation between stirring speed and polydispersity index. Thus, the result of the Mann–Kendall trend test confirms that there is no trend or correlation between stirring speed and polydispersity index. From the *τ* and *S* values for temperature and core size (nm) and zeta potential (mV), it is observed that core size exhibits a stronger trend (decrease) with increasing temperature than zeta potential. The reason for the null hypothesis observation of hydrodynamic diameter (nm), which is contradictory to what is observed from conventional graph plotting, is perhaps the lack of adequate data. The Mann–Kendall trend test data for stirring speed (rpm) and core size (nm), hydrodynamic diameter (nm), zeta-potential (mV) and polydispersity index PDI is summarized in Table 3.

**Table 3:** Mann–Kendall trend test data for correlation between speed (rpm) and core size (nm), hydrodynamic diameter (nm), zeta-potential (mV) and polydispersity index (PDI)

Parameter	Core size (nm)	Hydrodynamic diameter (nm)	Zeta potential (mV)	Polydispersity index (PDI)
Kendall's tau	−0.563	−0.600	−0.534	0.067
*S*	−76	−9	−35	1
Var (*S*)	587.333	28.333	211.667	28.333
***p*-Value (two-tailed)**	**0.002**	**0.133**	**0.019**	**1.000**
*α*	0.05	0.05	0.05	0.05

#### Sen's slope analysis for influence of stirring speed

From the Mann–Kendall trend test, it has been observed that the core size (nm) and zeta potential (mV) exhibit a significant decreasing trend with increasing temperature. The decreasing trend is further confirmed using Sen's slope estimator, where the core size (nm) *vs.* temperature (°C) graph yielded a Sen's slope of −0.075, whereby zeta-potential (mV) *vs.* temperature (°C) graph yielded a Sen's slope of −0.067. The sign of Sen's slope confirms the decreasing trend. In contrast, core size (nm) shows a more abrupt decreasing trend according to Sen's slope estimation, which is a more reliable quantification of the increasing/decreasing trend. Overall, the Sen's slope test data is summarized in Table 4.

**Table 4:** Sen's slope analysis data for correlation between speed (rpm) and core size (nm), hydrodynamic diameter (nm), zeta-potential (mV) and polydispersity index (PDI)

Parameter	Core size (nm)	Hydrodynamic diameter (nm)	Zeta potential (mV)	Polydispersity index (PDI)
Sen's slope	−0.075	−0.298	−0.067	0.001
Lower bound (95%)	−0.170	−0.545	−0.131	−0.003
Upper bound (95%)	−0.022	0.200	0.024	0.000

**Fig. 21:**
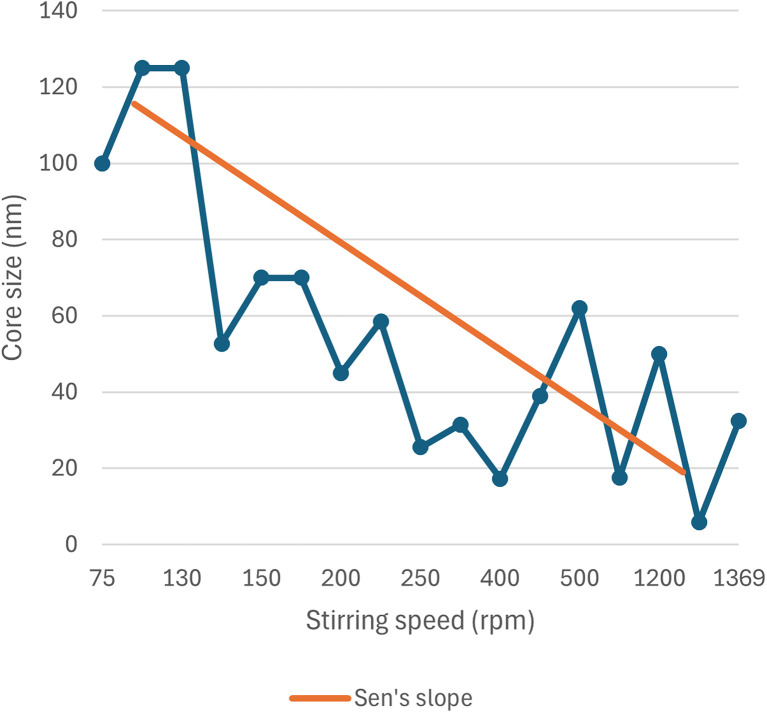
Correlation between stirring speed (rpm) and core size (nm) using Mann–Kendall trend test.

**Fig. 22:**
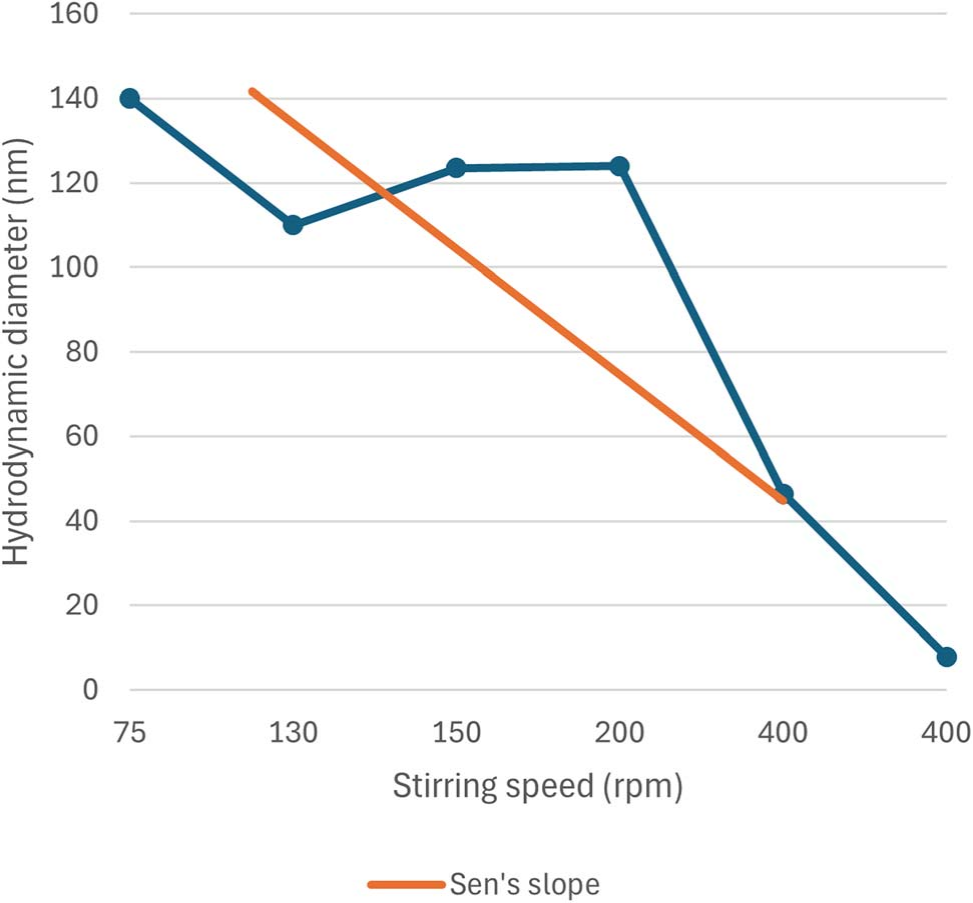
Correlation between stirring speed (rpm) and hydrodynamic diameter (nm) using Mann–Kendall trend test.

**Fig. 23:**
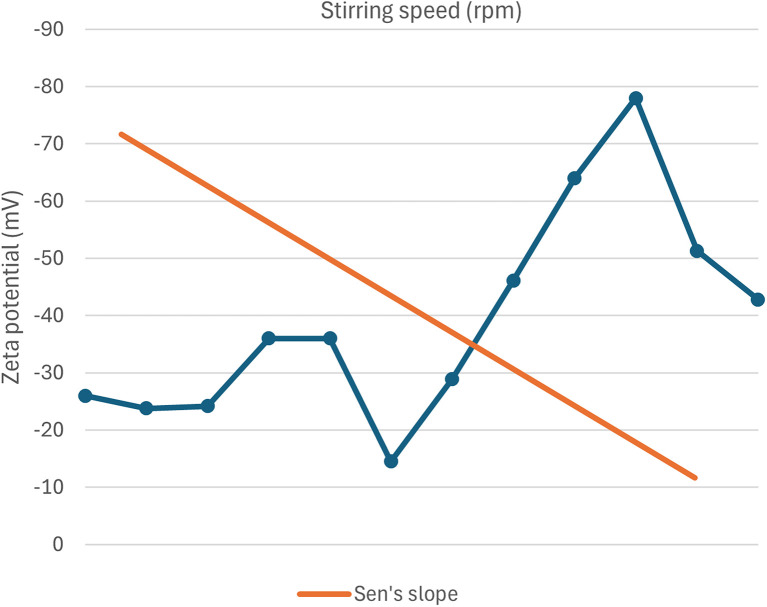
Correlation between speed (rpm) and zeta potential (mV) using Mann–Kendall trend test.

**Fig. 24:**
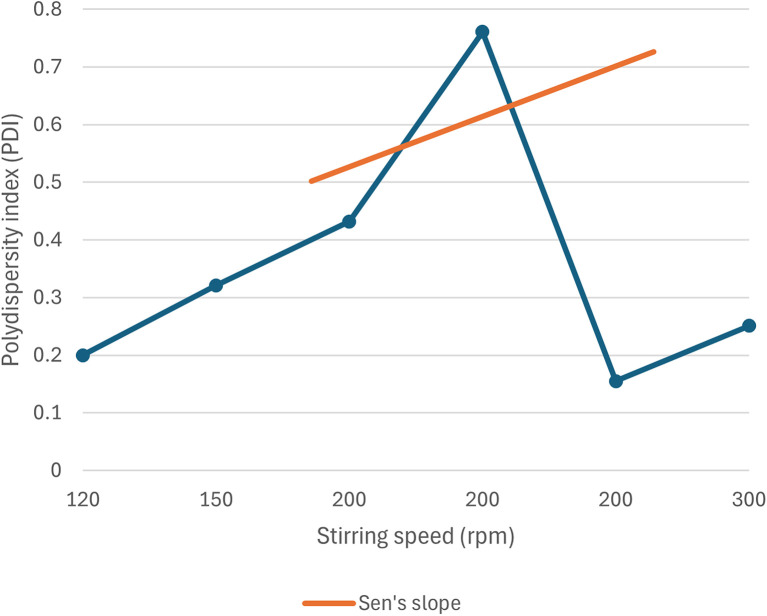
Correlation between speed (rpm) and polydispersity index using Mann–Kendall trend test.

The correlations between stirring speed (rpm) and core size (nm), hydrodynamic diameter (nm), zeta potential (mV), and polydispersity index (PDI) are plotted in Fig. 21–24.

#### ANOVA for stirring speed

One-way ANOVA for stirring speed (independent variable) and core size (dependent variable) rendered an *F*-statistic value of 8.468 and Pr (>*F*) value of 0.00828, which indicates the null hypotheses can be rejected as the Pr (>*F*) value is significantly greater than the significance value (0.05). Thus, the correlation between stirring speed and core size is statistically significant.

One-way ANOVA for stirring speed and zeta potential rendered an *F*-statistic value of 8.163 and Pr (>*F*) value of 0.0302, which is less than the significance value (0.05), thus null hypotheses can be rejected, and the statistical correlation between stirring speed and zeta-potential is significant.

### Influence of reaction time and incubation period

**Fig. 25:**
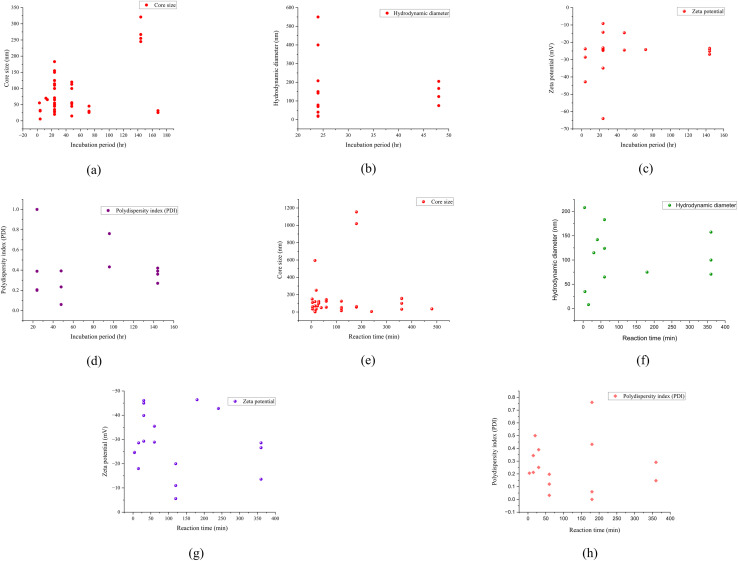
Randomness and scattered nature of impact of incubation period (hr) on (a) core size (nm), (b) hydrodynamic diameter (nm), (c) zeta potential (mV), and (d) polydispersity index (PDI). Randomness and scattered nature of impact of reaction time (min) on (e) core size (nm) (f) hydrodynamic diameter (nm) (g) zeta potential (mV), and (h) polydispersity index (PDI).

Both the reaction time and incubation period have a significant impact on the resulting green synthesized SeNp. The nanoparticles may agglomerate over time or shrink, resulting in a smaller particle size. This property primarily depends on the concentration of plant extracts and the phytochemicals present in them. Thus, no significant trend was observed with the influence of reaction time on the characteristics of the SeNp. The influence of reaction time and incubation period on the green synthesis of SeNp from many different plant extracts (available in SI) is graphically plotted in Fig. 25.

#### Mann–Kendall trend test for influence of reaction time

The Mann–Kendall trend test for reaction time and core size implied a *p*-value (two-tailed) of 0.514, reaction time and hydrodynamic diameter yielded a *p*-value (two-tailed) of 0.945, reaction time and zeta-potential rendered a *p*-value (two-tailed) of 0.834, and reaction time and polydispersity index rendered *p*-value (two-tailed) of 0.822. All the *p*-values (two-tailed) observed are higher than 0.05 (null hypothesis) and support the observation from conventional graph plotting (no trend, scattered data). The Mann–Kendall trend test data for reaction time (min) and core size (nm), hydrodynamic diameter (nm), zeta-potential (mV) and polydispersity index PDI is summarized in Table 5.

**Table 5:** Mann–Kendall trend test data for correlation between reaction time (min) and core size (nm), hydrodynamic diameter (nm), zeta-potential (mV) and polydispersity index (PDI)

Parameter	Core size (nm)	Hydrodynamic diameter (nm)	Zeta potential (mV)	Polydispersity index (PDI)
Kendall's tau	−0.080	−0.030	0.041	−0.050
*S*	−45	−2	7	−6
Var (*S*)	4548.333	212.667	815.000	493.333
***p*** **-Value (two-tailed)**	**0.514**	**0.945**	**0.834**	**0.822**
*α*	0.05	0.05	0.05	0.05

#### Sen's slope analysis for influence of reaction time

From the Mann–Kendall Trend test, no significant trend was observed between reaction time (min) and the characteristic parameters. This observation is further validated using Sen's slope estimator, where a negligible value of Sen's slope in every case proposes the presence of no trend. Sen's slope estimation data is summarized in Table 6.

**Table 6:** Sen's slope analysis data for correlation between reaction time (min) and core size (nm), hydrodynamic diameter (nm), zeta-potential (mV) and polydispersity index (PDI)

Parameter	Core size (nm)	Hydrodynamic diameter (nm)	Zeta potential (mV)	Polydispersity index (PDI)
Sen's slope	−0.047	0.034	0.006	0.000
Lower bound (95%)	−0.174	−0.277	−0.041	0.001
Upper bound (95%)	0.199	0.458	0.110	0.003

#### Mann–Kendall trend test for influence of incubation period

The Mann–Kendall trend test for incubation period and core size implied a *p*-value (two-tailed) of 0.381, incubation period and hydrodynamic diameter yielded *p*-value (two-tailed) of 0.584, incubation period and zeta-potential rendered a *p*-value (two-tailed) of 0.649, and incubation period and polydispersity index rendered *p*-value (two-tailed) of 0.428. All the *p*-values (two-tailed) observed are higher than 0.05 (null hypothesis) and support the observation from conventional graph plotting (no trend, scattered data). The Mann–Kendall trend test data for incubation period (hr.) and core size (nm), hydrodynamic diameter (nm), zeta-potential (mV) and polydispersity index PDI is summarized in Table 7.

**Table 7:** Mann–Kendall trend test data for correlation between incubation period (hr.) and core size (nm), hydrodynamic diameter (nm), zeta potential (mV) and polydispersity index (PDI)

Parameter	Core size (nm)	Hydrodynamic diameter (nm)	Zeta potential (mV)	Polydispersity index (PDI)
Kendall's tau	0.097	0.121	0.085	0.179
*S*	79	11	13	14
Var (*S*)	7923.667	333.667	697.000	268.667
*p*-Value (two-tailed)	0.381	0.584	0.649	0.428
*α*	0.05	0.05	0.05	0.05

#### Sen's slope analysis for influence of incubation period

From the Mann–Kendall trend test, no significant trend was observed between reaction time (min) and the characteristic parameters. Sen's slope value for core size (nm) and hydrodynamic diameter was observed to be 0.199 and 1.248, respectively, which is not negligible. However, as evident from the Mann–Kendall trend test, the null hypothesis cannot be rejected, and thus, the assumption from Sen's slope value is neglected. The data observed from Sen's slope estimation is summarized in Table 8.

**Table 8:** Sen's slope analysis data for correlation between incubation period (hr.) and core size (nm), hydrodynamic diameter (nm), zeta-potential (mV) and polydispersity index (PDI)

Parameter	Core size (nm)	Hydrodynamic diameter (nm)	Zeta potential (mV)	Polydispersity index (PDI)
Sen's slope	0.199	1.248	0.005	0.000
Lower bound (95%)	0.271	3.479	−0.002	0.000
Upper bound (95%)	2.034	0.000	0.643	0.008

### Applications of green synthesized SeNp

The application field of green-synthesized SeNp is diverse, ranging from biomedical applications to semiconductors; the application of SeNp is increasing daily. A particular advantage of synthesizing SeNp from plant extracts is that the plant extract acts as a capping agent, which is phenolic mainly and thus serves as a basis for medical applications. The overall applications of green-synthesized SeNp are schematically summarized in Fig. 27.

#### Biomedical applications

##### Anti-cancer activity

The anticancer activity of SeNp is attributed to its ability to destroy cancer cells, inhibit angiogenesis, and maintain healthy cells in a state of homeostasis.^95^ A common risk of cancer is due to the presence of arsenic (As) in drinking water. In one study, *Terminalia arjuna* (TA) leaf extract coated SeNp has been reported to inhibit the generation of As(iii) – activated ROS, which increases arsenic induced cell death.^96^ Fenugreek seed extract-mediated green synthesized SeNp shows excellent anticancer activity against the MCF – 7 breast cancer line, where it was conjugated with doxorubicin and increased the anticancer activity to a large extent.^41^

**Fig. 26:**
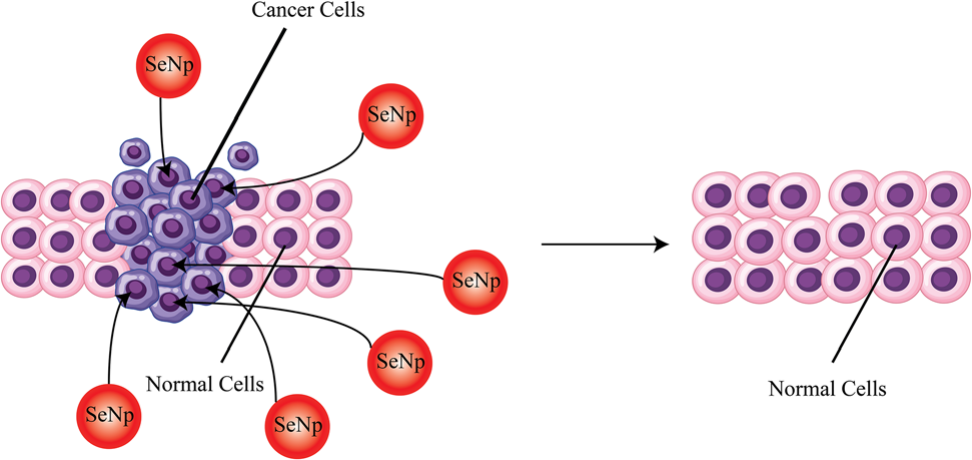
A simplified representation of the anti-cancer activity of SeNp.

In one study, *Ephedra aphylla*-mediated green synthesized SeNp demonstrated excellent cytotoxic effect against HepG2, HTC- 116 and HeLa cell lines.^97^ Cytotoxic activity against the A549 lung cancer cell line is also reported in another study, where the SeNp was encapsulated with *Enicostema axillare* extract.^98^ Hawthorn fruit extract-mediated SeNp demonstrated anticancer activity against the HepG2 cell line.^99^ Java tea extract-mediated green synthesized SeNp has been reported to be cytotoxic against the L6 cell line.^58^ In another study, garlic extract-mediated SeNp has been reported to directly interact with DNA and function as a cancer preventive agent.^100^ Aloe vera leaf extract-mediated green synthesized SeNp has been reported to be effective as a chemo-preventive agent in another study.^101^ The anti-cancer activity of SeNp synthesized using *Averrhoa carambola* leaf extract was assessed by running anti-ROS study, and the result has been reported to be satisfactory.^102^*Hibiscus esculentus* L. extract-mediated green synthesised SeNp was assessed for its anticancer activity in another study, which was conducted on human gastric cancer (AGS), human breast adenocarcinoma (MCF-7), human non-tumorigenic lung epithelial cell line (Beas) cells and the results were satisfactory.^103^ In another article, *Asteriscus graveolens* leaf extract was employed as the clean reducing agent, where the synthesised SeNp showed higher inhibition against HepG2 cell proliferation and induced high DNA damage by arresting cell cycle phases.^104^ The prevention of cancer by SeNp is schematically presented in Fig. 26.

**Fig. 27:**
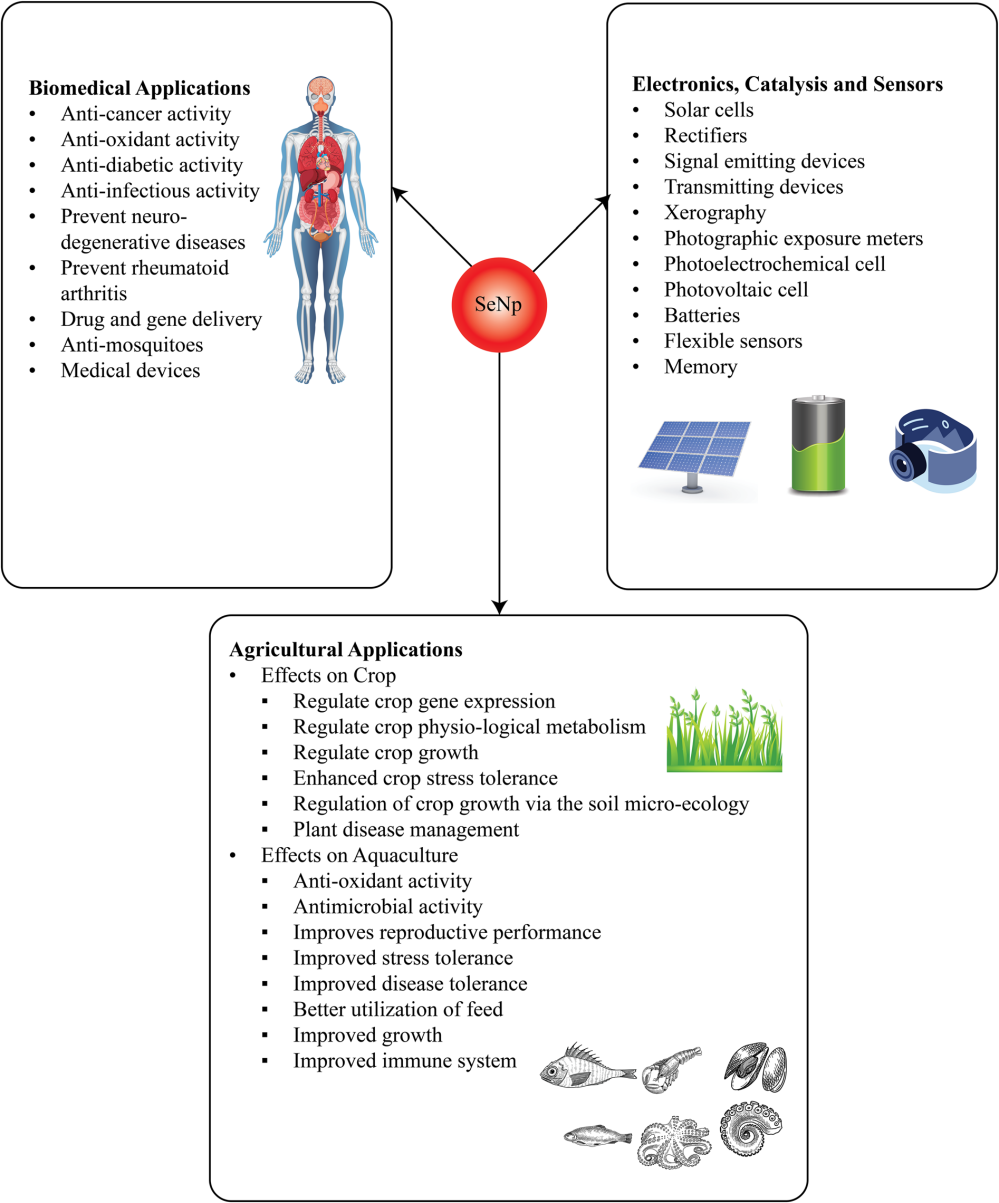
An overview of applications of SeNp.

##### Antioxidant activity

The plant extract-mediated SeNp is encapsulated chiefly with phytochemical compounds from the plant extracts, which play a key role in the antioxidant activity of the SeNp. SeNp can reduce the concentration of free radicals to prevent oxidative damage to DNA.^105^ The evaluation of the antioxidant potential of SeNp in the literature is performed using various assays, including 2,2′-diphenyl hydroxyl (DPPH) and 2,2′-casino-bis (3-ethylbenzothiazo line-6-sulphonic acid) (ABTS) as well as ferric reducing antioxidant power (FRAP) using standard procedures.^83^ In one study, the antioxidant capacity of SeNp was assessed by DPPH and ABTS free radical scavenging assays.^106^ It rendered satisfactory results, which may be due to the antioxidant action of encapsulating *Emblica officinalis* extract or may be due to SeNp alone, as SeNp governs the upregulation of selenoenzymes like glutathione peroxidase and assists in protecting cells and tissues under *in vivo* conditions from free radicals.^107^*Averrhoa carambola* leaf extract-mediated green synthesized SeNp has been reported to demonstrate antioxidant properties which DPPH and hydroxyl radical scavenging activity.^102^

**Fig. 28:**
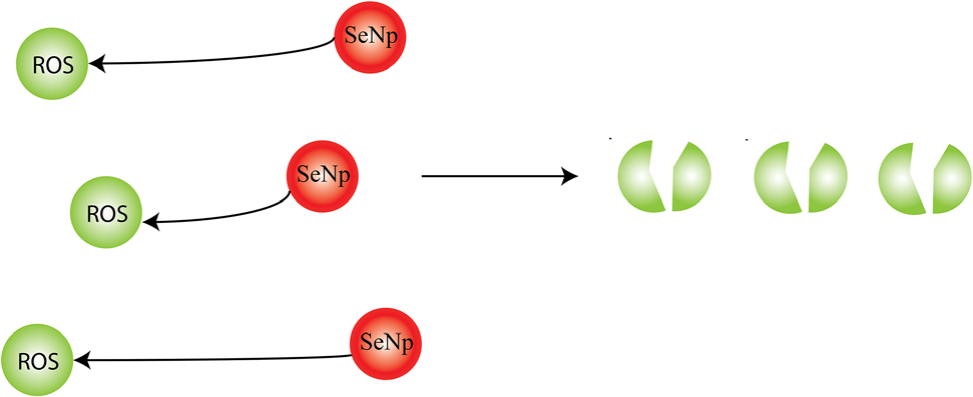
An illustration representing the antioxidant activity of SeNp.

Antioxidant capacity of SeNp synthesized from the extracts of *Hibiscus sabdariffa*,^88^ Cacao bean shell,^108^*Allium sativum*,^109^ Bee propolis,^110^*Camellia sinensis* (L) Kuntze Leaves,^111^ Broccoli,^112^ Herbal,^113^*Ocimum tenuiflorum* L,^114^ Green Coffee Beans,^115^*Cannabis sativa*,^116^*Delonix regia*,^60^*Nerium oleander*,^60^*Diospyros montana* Leaf,^40^ Pomegranate peel,^117^ and *Moringa oleifera* are also reported in the literature.^54^ As of our literature survey, plant extract-based SeNp has almost spherical morphology in all cases, and hollow spherical SeNp is known to show better anti-oxidative properties.^12,17,118^ Thus, plant extract-mediated SeNp can be adopted in cases where significant anti-oxidative performance is desired. The phenomenon of antioxidant activity of SeNp is schematically presented in Fig. 28.

##### Anti-diabetic activity

SeNp can significantly perform as an anti-diabetic agent due to its ability to mimic the physiological functions of the hormone insulin.^95^ SeNp has been reported in the literature to effectively regulate the blood sugar levels.^119^ Hypoglycaemia is the most noticeable outcome of diabetes mellitus, which promotes oxidative stress by overproduction of reactive oxygen species (ROS).^120^ Overproduction of ROS in diabetes contributes to the development of chronic diabetic lesions in organs such as the liver and kidney.^121^ The mechanism of action of SeNp in anti-diabetic activity is complex and includes numerous inter-related pathways.^122^ In one study, the anti-diabetic performance of SeNp has been reported to be due to the antioxidant capacity of SeNp, as it destroys the ROS, which is responsible for diabetes mellitus.^123^ In another study, the mechanism of anti-diabetic action of SeNp was tried to be understood by using a combination of *Catathelasma ventricosum* polysaccharide (CVPs)-SeNp and VE which inhibits the production of MDA (methane dicarboxylic aldehyde), a marker of oxidative damage, but more chemical and pharmacological research is needed to understand the anti-diabetic actions of SeNp fully.^124^ SeNp loaded with insulin (INS-SeNps) can be delivered orally to treat diabetes in mice, as addressed in another study.^125^ the enhancing activity of hyperglycaemia and hyperlipidaemia in STZ-induced diabetes in mice when SeNp is combined with insulin is also reported in another study^126^ The extracts of mulberry leaf and *Pueraria lobata* (MPE) coated SeNp exert a hyperglycaemic effect that can also be employed to treat diabetes mellitus.^127^

##### Anti-infectious agent

When microorganisms are intricately linked to a variety of inflammatory and immunological responses through any means, then infectious disorders occur.^122^ Infections can occur by a range of microorganisms, such as bacteria, viruses, fungi, and parasites. Plant extract-mediated green-synthesised SeNp exhibits significant antibacterial, antiviral, antifungal, and anti-parasitic activities. Negatively charged SeNPs have been reported to be more effective in inhibiting the growth of Gram-positive bacteria; thus, research is now more focused on positively charged SeNPs for antibacterial activity.^128^ In another study, chitosan-PVA scaffolds were loaded with both SeNp and AgNp to assess their ability to destroy the bacterial cell wall, whereby SeNp demonstrated better cytocompatibility and good antibacterial properties.^129^ Lysozymes are the enzymes in the body that fight pathogens. A synergy between SeNp and lysozyme boosts the antibacterial activities of lysozymes, and they are also proven to promote the antibacterial activities of lysozyme.^130,131^ A study reported that SeNp can be effectively used to inhibit the action of *Mycobacterium tuberculosis* (*M. tuberculosis*), which causes tuberculosis and often becomes resistant to drugs.^132^ In another study, antibacterial activities against many bacteria were assessed using organ selenium compounds, and the results exhibited extraordinary antibacterial activity.^133,134^ Effective inhibition properties against *Escherichia coli* and *Staphylococcus aureus* were reported when incubated with lysozyme in the form of a monohybrid system.^131^ In another study, irreversible damage to the *Staphylococcus aureus* bacterial cell wall membrane was observed by a nanocomposite surface conjugating quercetin (Qu) and acetylcholine (Ach) selenium nanoparticles (Qu–Ach@SeNPs).^135^ The antiviral potential of SeNp has been addressed in other studies, where it has been reported that SeNp slows the course of dengue virus infection.^136,137^ SeNp also have the potential to be used in direct antibacterial and antiviral therapy.^138,139^*Candida albicans* are a species of pathogenic yeast that is the source of many infectious diseases. Chitosan-SeNp has been reported to be effective against *Candida albicans*.^140^ SeNp is incorporated into clothes to achieve antibacterial and antifungal properties. A SeNp modified with biogenic polysaccharide–protein (PSP) complexes collected from *Pleurotus tuber-regium* deposited on a fabric demonstrated antibacterial properties against *S. aureus* and *Trichophyton rubrum* bacteria.^141^

**Fig. 29:**
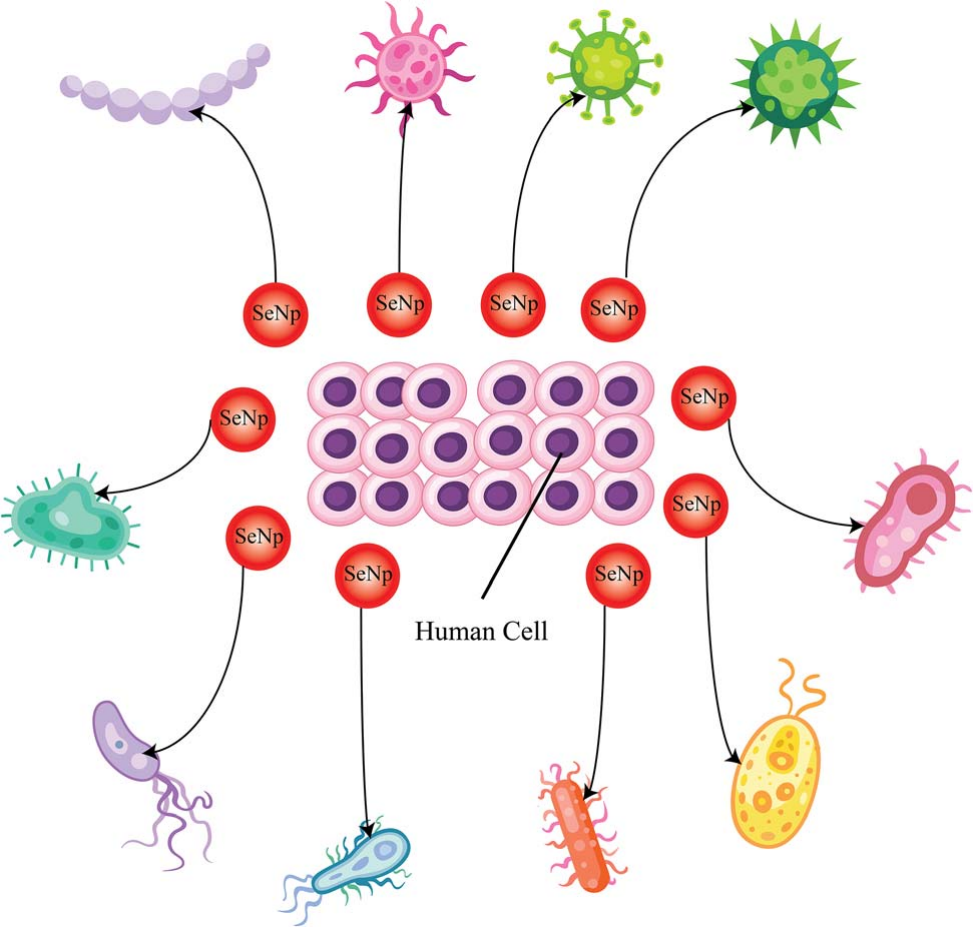
A general schematic of SeNp functioning as an anti-infectious agent.

Patients with reduced immunity to nystatin-immune Candida species may find SeNp to be a suitable alternative for providing antifungal action.^16^ SeNp bolsters the potency of *Echinococcus granulosus* by imposing suicidal activities.^142^*Oligoporus pelliculosus* is responsible for the rotting of wood, and green synthesized SeNp from plant extracts have demonstrated significant anti-fungal capacity against it.^143^ Thus, biosynthesized SeNp with a particular particle size range is effective against *Leishmania* parasitic diseases. A general schematic of SeNp functioning as an anti-infectious agent is illustrated in Fig. 29.

##### Prevention of neurodegenerative diseases

Reactive oxygen species (ROS) and reactive nitrogen species (RNS) are free radical species, both of which are produced in the brain. ROS and RNS produce oxidative stress and lead to many neurodegenerative diseases, such as Parkinson's disease, Alzheimer's disease, Huntington's disease, epilepsy, cerebral ischemia, and traumatic brain injury. SeNp, being an effective antioxidant, breaks down the ROS and RNS to reduce oxidative stress and thus prevent neurodegenerative diseases. Patients suffering from brain diseases such as Alzheimer's disease and Huntington's disease are reported to have a deficiency of selenium, which leads to brain dysfunction and detriment.^14,144^ Selenium therapy has been shown to lower oxidative stress in the brain of Alzheimer's disease experimental animal models.^122^ Thus, SeNp is expected to deter the rate of neurodegenerative disease.

##### Prevention of rheumatoid arthritis

Rheumatoid arthritis is a chronic, inflammatory, autoimmune disorder that can affect the joints, as well as the skin, eyes, lungs, heart, and blood vessels, because the immune system mistakenly attacks the body's tissues. Almost all the therapeutic applications available for rheumatoid arthritis have severe side effects. Nanomedicine is an emerging field for the treatment of rheumatoid arthritis.^145^ SeNPs dispersed in 1% phytochemical coumaric acid are reported to heal rheumatoid arthritis (RA) in rat models.^146^

**Fig. 30:**
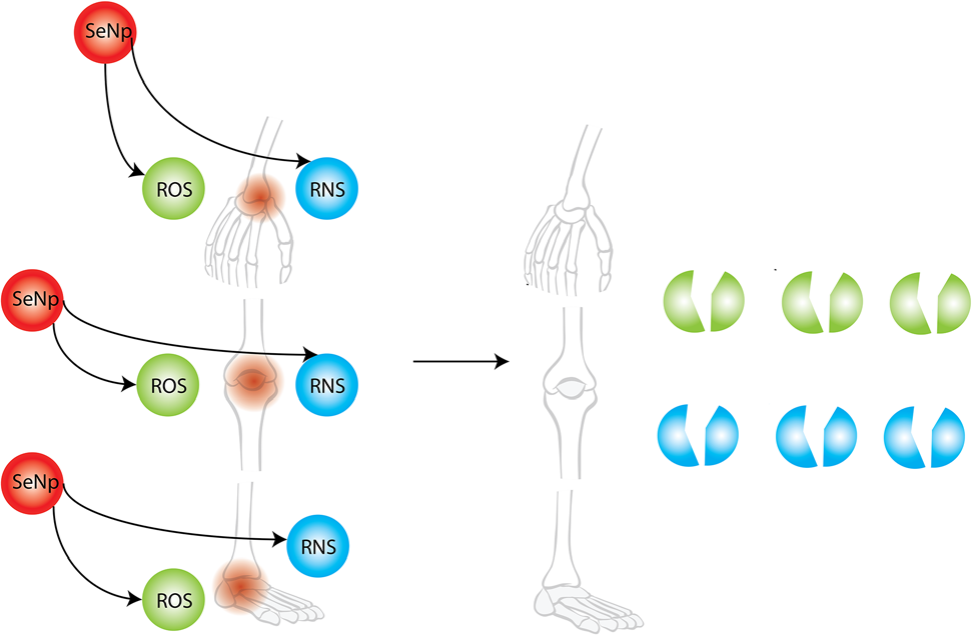
Prevention of rheumatoid arthritis by green synthesized SeNp.

Both inflammation and oxidative stress play crucial roles in the development of rheumatoid arthritis.^147,148^ Owing to the anti-inflammatory and antioxidant capability of SeNp, it can be used as a treatment strategy to cure rheumatoid arthritis. The general schematic of SeNp preventing rheumatoid arthritis is represented in Fig. 30.

##### Drug and gene delivery

Nanoparticles are abruptly used in the present day for the loading and delivery of drugs and genes due to their specificity to the sites of action within the body. SeNp, owing to its biocompatible nature and unique properties, is emerging as a new technology for drug and gene delivery. SeNp has distinctive features as nano-carriers, such as the reduction of drug volume, restriction of drug exposure to healthy cells and tissues, distribution of the drug in solid colloidal form, improving the solubility of hydrophobic materials for parenteral administration, and amplifying the stability of therapeutic agents, which help them to be used as drug carriers.^128^ Propylene-oxide modified ruthenium complexes and ethylene oxide copolymer, considered inorganic therapeutics drugs in cancer treatment, has been reported to be delivered using SeNp.^149^ Doxorubicin-cisplatin causes apoptosis in the mammalian breast cancer cell, and the attempt to deliver this drug combination using SeNp was addressed in another study.^150^ In another application, Se/SiO_2_/FA/CuS/DOX nanocomposite has been reported to perform photothermal and chemotherapy for cancer treatment.^151^ SeNps induce apoptosis *via* intrinsic and extrinsic pathways involving p53 activation and caspase-mediated routes.^122^ SeNp has also been reported to cure the thyroid damage that is induced by Cr(vi).^152^ In another study, the efficiency of irinotecan with or without SeNp has been assessed, and the result showed that when combined with SeNp, greater toxicity towards the HCT-8 cancer cell line is observed.^153^ Polyamidoamine and polyamidoamine–dendrimer–modified SeNp delivered siRNA and cisplatin to the tumour, where the cell apoptosis occurred through PI3K/Akt/mTOR and MAPK/ERK pathways.^154^ Crocin-conjugated PEG505 SeNp exhibited potential hemocompatibility.^155^ Curcumin-loaded SeNp exhibited cancer treatment activities against Ehrlich's ascites carcinoma in a mouse model through apoptosis.^156^

Gene therapy involves the incorporation of a therapeutic agent into the genetic material to resist disease progression and proliferation.^128^ In one study, siRNA packed inside polyethyleneimine-modified SeNp was delivered to the cancer cells to destroy them.^157^ Chitosan-coated SeNp is used for mRNA delivery to tumour cells, exhibiting its potential in tumour vaccination and immunotherapy.^122^ Thus, SeNp has a high potential for drug and gene delivery in the field of nanomedicine.

##### Anti-mosquitoes

A unique but interesting application of SeNp is its insecticidal potential against mosquito vectors. Mosquitoes are the primary cause of many diseases affecting humanity, including chikungunya, dengue, and malaria. In one study, the *Clausena dentata* plant leaf extract-mediated green synthesised SeNp was assessed for its anti-mosquito activity. A larvicidal bioassay was conducted on the larvae of *Anopheles stephensi*, *Culex quinquefasciatus*, and *Aedes aegypti*, and the results indicated its potential to be used as an anti-mosquito agent.^137^

**Fig. 31:**
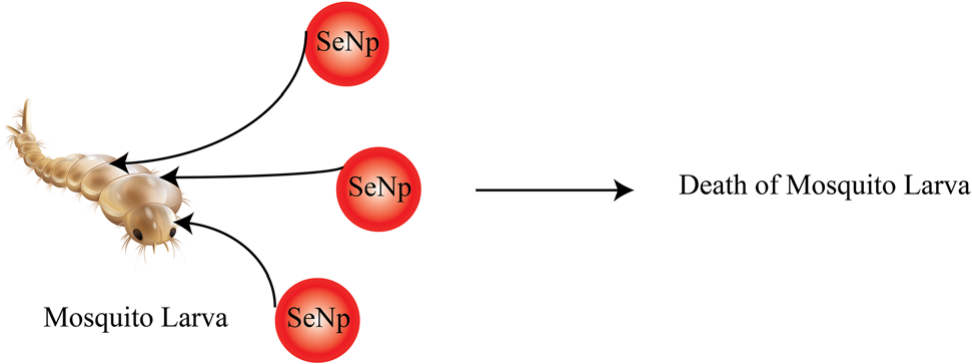
Mosquitocidal action of SeNp.

In another study, *Nilgirianthus ciliates* leaf extracts mediated green-synthesised SeNp was assessed for its pesticidal activity against *Aedes aegypti*, a species particularly responsible for dengue outbreaks.^158^ Mosquitoes were collected, and eggs were taken to the laboratory. Larval toxicity assay and histopathological assay were conducted, which rendered satisfactory results for it to function as an anti-mosquito agent. The mosquitocidal activity of green synthesized SeNp from *Ceropegia bulbosa Roxb* was reported in another study, and the larvicidal activity conducted on *Ae. Albopictus* yielded satisfactory results, making it an effective anti-mosquito agent.^159^ The common anti-mosquito agents are usually detrimental to health and the environment; thus, the SeNp can work as an eco-friendly alternative. The phenomenon of mosquito larvae being killed by SeNp is schematically presented in Fig. 31.

##### Medical devices

Medical devices are being used in the present world to extend life, with the most prominent uses including diagnosis, monitoring, and treatment of injuries or diseases.^160^ Green synthesized SeNp from plant extracts is known to exhibit antimicrobial properties. Polymeric medical devices are coated with green synthesized SeNp to exhibit antimicrobial action, which is previously reported in the literature.^161^ To introduce antibacterial properties to poly(ether ether ketone) (PEEK) based medical devices such as orthopaedic instruments, spinal fusion devices, and segments in dialysis equipment, they are coated with red SeNp.^162^ PVC is commonly used as a medical tube substrate, which is prone to bacterial infection. The incorporation of SeNp significantly enhanced the antibacterial activity and anti-biofilm formation in PVA substrate medical devices.^163^ In another study, anti-biofilm capability of SeNp was assessed as an alternative to AgNp, as Ag is not a biocompatible material, and the results were quite satisfactory.^164^ Polycarbonate medical devices were coated with SeNp, where the particles were 50–100 nm in diameter and well distributed in the polycarbonate surface, subsequently exhibiting anti-bacterial properties.^165^

##### Electronics, catalysis and sensors

Selenium nanoparticles have recently gained widespread attention in electronic sectors due to their unique properties. Green-synthesised SeNp has been reported to be used in solar cells, rectifiers, signal-emitting devices, transmitting devices, xerography, and photographic exposure meters.^166^ The work function of SeNp is higher than the work function of many metals, which facilitated the use of SeNp in memory devices.^167^ The memory behaviour of SeNp is found to be unique compared to commonly used semiconductor and metal species. The application of SeNp as a charge storage element for flexible, semi-transparent memory devices is reported in the literature.^168^ SeNp has been reported to have a band gap of ∼1.7 eV.^169^ SeNp has been previously reported to exhibit photocatalytic and photoconductive activities. Owing to the facile hole propagation that occurs along the *c*-axis of trigonal SeNp with a hexagonal crystal structure, SeNp is photoconductive.^170^ SeNp-decorated silicon nanowires with enhanced liquid-junction photoelectrochemical solar cell performance were studied in another study, where SeNp is particularly cost-effective and enhances the power conversion efficiency (PCE) by relying on unique mechanisms.^171^ SeNp has abundant applications in the field of photocatalysis and energy storage and conversion, and SeNp is commonly employed as a dopant in intrinsic material owing to its low toxicity, narrow bandgap structure, and high surface area.^172^ In another study, the thermoelectric properties of SeNp coated with PEDOT: PSS were assessed, and the result showed that the thermal conductivity was higher than either SeNp or that of the pure polymer.^173^ Owing to the unique properties of SeNp, such as anisotropic structure, quantum confinement effects, large surface areas, and optical, electrical, optoelectronic, electrochemical, photovoltaic and piezoelectric properties, SeNp is currently being used to design flexible and wearable electronics.^174^ One of the most groundbreaking applications of SeNp in the energy sector is incorporating SeNp in the electrode, replacing conventional electrodes to protect the environment and also increase efficiency. Li–S or Li–Na batteries are emerging technologies in the present world owing to their high specific capacities and energy densities. Li–Se and Na–Se batteries were designed by incorporating selenium into a porous 3D interconnected network of carbon nanofibers (CNF) for superior electrochemical performance.^175^ In another study, photosensitive SeNp was tethered to silicon nanowires for solar energy conversion, where the incorporation of SeNp increased the efficiency of solar cells from 4.91% to 43%.^176^ The application of SeNp in optical devices, photovoltaic solar cells, photo-assisted direct methanol fuel cells, catalysis, and chemical sensors have been elaborately discussed in previous study, where it acts as a direct catalyst in photo-assisted direct methanol fuel cells.^177^ In another study, SeNp has been reported to be employed as nano sensors, where AC electrokinetic manipulation of SeNp has been done.^178^ PVA/CMC blend for food packaging and electrochemical battery applications doped with SeNp was studied in another study, where the electrical conductivity and antibacterial properties increased after doping with SeNp.^179^ SeNp has also been applied in photographic exposure meters, solar cells, electrical rectifiers, pressure and chemical sensors, xerography, and fuel cells.^180^ Methylene blue dye is a widely used dye that is a well-known carcinogen and detrimental to the environment. The degradation of methylene-blue dye by SeNp was reported in another study, whereby it was addressed that SeNp can degrade the methylene-blue dye when exposed to solar radiation, which was confirmed by a decrease in intensity of the characteristic methylene-blue dye peak in the sample.^181^ In another study, green synthesized SeNp from *Withania somnifera* leaves extract was assessed for its photocatalytic activities, where the results showed a positive response to the degradation of methylene blue dye.^182^ Photocatalytic activity was assessed using MB dye in another article, where SeNp was synthesized from *Elaeagnus indica* leaf extract.^183^ In another study, the photocatalytic activity of green synthesized SeNp from *Ceropegia bulbosa Roxb* extract was assessed by observing the degradation of MB with and without SeNp, and the sample dye loaded with SeNp exhibited faster degradation rates.^184^*Moringa oleifera* extract-mediated green synthesized SeNp was evaluated for its photocatalytic activity using the degradation of sunset yellow azo dye, where SeNp showed better efficiency in degrading sunset yellow azo dye both under solar and UV radiations.^185^ In another study, *Aspergillus terreus* mediated green synthesized SeNp was assessed for its photocatalytic activity by the degradation of green malachite dye, and significant degradation was observed.^186^

#### Agricultural applications

##### Effects on crop

SeNp has gained considerable attention in recent years due to its alleviation capacity against a variety of biological and abiotic stresses, including heavy metals, salinity, drought, and high temperatures, as well as the inhibition of pathogenic microorganisms.^187^ SeNp alone is often prone to agglomeration. To enhance the stability of SeNp, macromolecular substances such as polysaccharides, proteins, *etc.*, are added as templates.^188^ Selenium nanoparticles have been reported to alleviate abiotic stresses when applied to crops.^189^ When SeNp is uptake by plants, the SeNp is converted into organic selenium in crops, which promotes an enhanced reactive oxygen species (ROS) removal system and inhibits the adsorption of heavy metals in plants, promoting antipathogenic microbial agents and abiotic stress.^190^ SeNp can regulate the growth of crops by increasing total flavonoids, total antioxidant capacity, total phenols and vitamin C levels, as reported in the previous study.^191^ SeNp can regulate crop physiological metabolism if it is applied *via* foliage spraying or soil irrigation. The effect of SeNp when applied in soil treatment is addressed in another study, where a significant increase in the grain yield was observed when the bioconcentration of SeNp was 1–10 mg kg^−1^.^192^ SeNp has been reported to effectively regulate crop-gene expression in another study.^193^ Green synthesized red SeNp have scavenging effects of various free radicals, whereas grey selenium and black selenium are not biologically active.^14,118^ In another study, the growth of antipathogenic microorganisms was shown to increase with increasing SeNp concentration.^194,195^ The effect of SeNp on two important antipathogenic soil microorganisms, *Bacillus* sp. and *Escherichia coli*, was evaluated in another study.^196^ It was found that SeNp did not enter these microorganisms, indicating lower toxicity and higher safety of SeNp. Selenium nanoparticles have also been proven to enhance crop stress tolerance. In one study,^197^ incorporating SeNp has been reported to reduce the effect of NaCl stress on the growth of tomato crops.^198^ In another study, SeNp has been proven to repair penthiopyrad (Pen) stress.^199^ Articles in the literature provide data on SeNp being effective as a plant fertilizer. In one study, production and use of SeNp as fertilizers were assessed, and the results showed enhanced development of the plant supplemented with SeNp.^200^ In another study, green-synthesised SeNp from *Cassia javanica* flower extract was found to be effective in increasing plant growth from the germination stage to the root growth, shoot growth, and vegetative stage.^201^ In another article, the application of green synthesized SeNp in plant disease management was addressed, where the SeNp acts directly on bacterial cells and fungal cells, inhibits the electron transport chain, which results in damage to proton efflux pump, destabilizes ribosomes, produces RO, and all this phenomenon eventually leads to cell death, which ensures plant disease management and prevents the formation of biofilm.^202^

##### Effects on aquaculture

Aquaculture is one of the fastest food-producing systems in the world, facing critical challenges such as disease outbreaks and environmental degradation. SeNp has been proven to have a positive effect on aquaculture. The capacity to offer antimicrobial, antioxidant, and growth-promoting properties, supporting the gut economy and digestive capacity in aquatic animals, along with higher bioavailability and the ability to traverse gut barriers, makes them candidates for aquafeed supplementation.^203^ Dietary supplement of SeNp depends on the species of the aquatic animal. The SeNp has been reported to affect growth activity, immune system activity, antioxidant activity, disease resistance, and stress tolerance in previous literature. A dietary supplement of 0.68/mg/kg/70 days is reported to increase the growth performance of *Tor putitora*.^204,205^ SeNp can boost the immune system activity by increasing the activities of T-cells and natural killer (NK) cells, as for the case of dietary supplementation of 1 mg/kg/45 days SeNp in *Pagrus major*.^206,207^ The antioxidant capability of SeNp is vital because the stress elements in the aquatic environment increase the production of oxide and peroxide radicals, which are detrimental to aquatic organisms. A dietary supplement of SeNp at 1 mg kg^−1^ is found to increase the antioxidant potential of *Oncorhynchus mykiss*.^208^ Inclusion of SeNp supplementation has been proven to increase the ability of aquatic animals to resist pathogens and infections, contributing to their well-being.^209^ Dietary incorporation of SeNp incorporated spirulina at a concentration of 5% during a 14 days feeding trial demonstrated a significant reduction in the mortality rate when *Lates calcarifer* were exposed to *Vibrio harveyi* infection.^203^ Stress tolerance activity of aquatic animals was also greatly enhanced by the incorporation of SeNp. At dietary supplements of 1–2 mg kg^−1^, the resistance of *Pangasianodon hypophthalmus* (*P. major*) against low salinity stress was reported to be improved.^210^

### Required purity levels of plant-mediated, green-synthesized selenium nanoparticles (SeNp) for diverse applications

The purity of plant extract-mediated, green-synthesized nanoparticles refers to the absence of toxic chemicals and the presence of natural phytochemicals that act as reducing and stabilizing agents during the synthesis process.^211^ Based on our literature survey, there is a lack of specific information on the required purity levels of green-synthesized selenium nanoparticles for different applications. However, based on the author's observations, required purity levels of green-synthesized selenium nanoparticles can be predicted, which are summarized in Table 9. High purity refers to low impurities (<30 ng g^−1^) and consistent particle size,^212^ while moderate purity refers to capping by phytochemical compounds (enhanced stability and bioactivity).^213^

**Table 9:** Required purity level of green-synthesized selenium nanoparticles with underlying reasons

Application	Required purity level	Reason
Biomedical	High	Stability and biocompatibility are crucial^214^
		Functionalization enhances efficacy and reduces toxicity^215^
		Purity affects targeted delivery and cytotoxicity^216^
Electronics, catalysis, and sensors	High	Purity impacts structural morphology and chemical composition^217^
		Stability and size consistency are important^217^
Agricultural	Moderate to high	Purity affects growth enhancement and antimicrobial activity^218^
		Stability and dispersion are important for effective application^219^

## Conclusion

Throughout this article, an attempt was made to devise any correlation between reaction parameters and the resulting properties in the green synthesis of selenium nanoparticles; the trends observed were further validated using the Mann–Kendall trend test, and the trend was quantified using Sen's slope estimator, the correlation was further validated using ANOVA, and the application of plant extract mediated green synthesized SeNp was addressed. An increase in temperature exhibited a decreasing trend in particle size and an increasing trend in zeta potential, which aligned with the theoretical basis. The Mann–Kendall trend test and Sen's slope estimation also rendered a parallel trend with a conventional graph linear fit. However, it showed a contradictory phenomenon to that observed by ANOVA, where temperature was found to have no statistically significant relationship with core size. However, it showed the same trend in the case of zeta potential. No trend was observed in the case of temperature and polydispersity index because other operational parameters dominate over this factor, which is further supported by the Mann–Kendall trend test and Sen's slope estimation. The increase in stirring speed showed a decreasing trend in the particle size and an increasing trend in the zeta-potential, which also coincides with the result obtained from the Mann–Kendall trend test, Sen's slope estimation, ANOVA test, and also matched with the theoretical basis. The higher Sen's slope value in the case of the influence of temperature on core size (−1) and zeta-potential (−0.205) compared to the influence of stirring speed on core size (−0.075) and zeta-potential (−0.067) indicates that temperature has a more significant effect on core size and zeta-potential. The influence of concentration (plant extract and precursor solution), their volume, and reaction time depicted very scattered results because they vary with the plant extracts used and the phytochemical constituents present in them, and this was also supported by the Mann–Kendall trend test and Sen's slope estimation. Exactly linear behaviour is not observed in any case because the reducing agent used, its concentration, precursor solution used, and its concentration, reaction time are co-related with the input parameters discussed in this paper to devise an order or trend of increasing/decreasing property. Thus, non-linear behaviour is expected in this case. However, this article will provide a general guideline on how the input parameters discussed relate to the characteristic properties of SeNp and their potential values that may be employed in future works. The application of SeNp in various fields of the modern world is elaborately discussed, which may help future enthusiasts interested in working with SeNp. There are numerous potential applications of green-synthesised SeNp due to its versatile properties, and further research in this area is likely to be conducted in the future. This article is expected to be of use to researchers in optimizing the process parameters to achieve the desired particle size and other properties for their specific applications. In the future, further research can be conducted on the simultaneous effect of reaction parameters on the characteristic properties of green-synthesized selenium nanoparticles, rather than a one-way approach, if an adequate data repository is available.

## Author contributions

Md. Saiful Islam: Conceptualization, data curation, formal analysis, investigation, methodology, resources, software, validation, visualization, writing-original draft, writing-review & editing. Sams Uddin Sams: Formal analysis, investigation, visualization, writing-original draft. Sadit Bihongo Malitha: Funding acquisition, investigation, methodology, project administration, supervision, validation, writing-review & editing. Md. Zahangir Alam: Project administration, supervision, validation, writing-review & editing.

## Conflicts of interest

There are no conflicts to declare.

## Supplementary Material

RA-015-D5RA03940A-s001

RA-015-D5RA03940A-s002

## Data Availability

The data supporting this article have been included as part of the supplementary information. The supplementary information file includes the essential tables for a comprehensive understanding of the trend analysis graphs. See DOI: https://doi.org/10.1039/d5ra03940a.
