# Supplementary material for: Trend analysis of reaction parameters and properties of plant-mediated green synthesized selenium nanoparticles using non-parametric statistical methods (Mann–Kendall trend test, Sen's slope estimator, ANOVA) and their applications in the modern world: a critical perspective
Source: RSC Adv. 2025 Aug 7;15(34):28155–80. doi: 10.1039/d5ra03940a (PMC12376871; doi:10.1039/d5ra03940a)
Supplement: RA-015-D5RA03940A-s001 [file RA-015-D5RA03940A-s001.pdf]

Supplementary Information (SI) for RSC Advances.
This journal is © The Royal Society of Chemistry 2025

**Table 1: Influence of Volume on particle size (nm), zeta potential (mV) and polydispersity index (PDI).**

| **Precursor solution** | Volume (PS) (mL) | Plant Extract | Volume (PE) (mL) | NP Core Size (nm) | Hydrodynamic diameter (nm) | Zeta Potential (mV) | Polydispersity Index (PDI) | References |
|---|---|---|---|---|---|---|---|---|
| **$Na_2SeO_3$** | 20 | *Aloe vera* leaf extract | 3.00 | 498 | - | - | - | [1] |
| | 20 | | 5.00 | 121 | | | | |
| | 27.07 | | 1.59 | 893 | | | | |
| | 20.00 | | 1.00 | 142 | | | | |
| | 12.92 | | 1.59 | 1315 | | | | |
| | 20.00 | | 3.00 | 831 | | | | |
| | 20.00 | | 3.00 | 741 | | | | |
| | 20.00 | | 3.00 | 742 | | | | |
| | 12.93 | | 4.41 | 923 | | | | |
| | 10.00 | | 3.00 | 3243 | | | | |
| | 30.00 | | 3.00 | 911 | | | | |
| | 27.07 | | 4.41 | 599 | | | | |
| | 20.00 | | 3.00 | 894 | | | | |
| | 13.33 | | 4.92 | 50 | 50 | -18 | 0.344 | |
| **$Na_2SeO_3$** | 32.22 | *Pelargonium zonale* leaf extract | 0.79 | 319 | - | - | - | [2] |
| | 40.00 | | 0.5 | 321 | | | | |
| | 57.67 | | 0.79 | 322 | | | | |
| | 57.67 | | 2.21 | 322 | | | | |
| | 40.00 | | 2.5 | 323 | | | | |
| | 15.00 | | 1.5 | 319 | | | | |
| | 40.00 | | 1.5 | 324 | | | | |
| | 40.00 | | 1.5 | 322 | | | | |
| | 40.00 | | 1.5 | 323 | | | | |
| | 40.00 | | 1.5 | 324 | | | | |
| | 40.00 | | 1.5 | 323 | | | | |
| | 65.00 | | 1.5 | 323 | | | | |
| | 22.32 | | 2.2 | 321 | | | | |
| | 15.00 | | 1.48 | 40-60 | 136 | -24.6 | | |

**Table 2: Influence of temperature on SeNp size**

| Precursor Solution | Plant Extract | Temperature (°C) | Core Size (nm) | Hydrodynamic diameter (nm) | References |
|---|---|---|---|---|---|
| **$H_2SeO_3$** | Roselle plant leaf extract | 20-22 | 35 | 20-50 | 3 |
| **$Na_2SeO_3$** | Lemon leaf extract (LLE) | 25 | 60-80 | 50 – 80 | 4 |
| **$Na_2SeO_3$** | Hawthorn fruit extract | 25 | 113 | - | 5 |
| **$Na_2SeO_3$** | Ginger fruit extract | 25 | 100-150 | - | 5 |
| **$H_2SeO_3$** | *W. somnifera* leaves extract | 25 | 40-90 | - | 6 |
| **$H_2SeO_3$** | *Trigonella foenum-graecum L* extract | 25 | 50-150 | - | 7 |
| **$Na_2SeO_3$** | *Cassia auriculata* extract | 25 | 10-20 | - | 8 |
| **$H_2SeO_3$** | *Orthosiphon stamineus* extract | 25 | 88-141 | - | 9 |
| **$H_2SeO_3$** | *Leucs lavandolifolia* extract | 25 | 56-75 | - | 10 |
| **$Na_2SeO_3$** | *Allium sativum* extract | 25 | 8-52 | - | 11 |
| **$NaHSeO_3$** | *Petroselinum crispum* extract | 25 | 10-300 | 400 | 12 |
| **$H_2SeO_3$** | *Astericus graveolens* extract | 25 | 20.06 | 20.17 | 13 |
| **Selenium Powder** | *Clausena dentata* plant leaf extract | 25 | 46.32-78.88 | - | 14 |
| **$H_2SeO_3$** | *Diospyros montana* leaf extract | 25 | 4-16 | - | 15 |
| **$SeS_2$** | *Ficus benghalensis* leaf extract | 25 | 20-140 | 45-95 | 16 |
| **$Na_2SeO_3$** | *Moringa Oleifera* extract | 25 | 23-35 | - | 17 |
| **$Na_2SeO_3$** | *Zingiber officinale* extract | 25 | 100 – 150 | 100 – 120 | 18 |
| **$Na_2SeO_3$** | *Emblica officinalis* fruit extract | 27 (±)2 | 15-40 | 20-60 | 19 |
| **$Na_2SeO_3$** | Cinnamon extract | 28 | 34.5-45 | - | 20 |
| **$SeO_2$** | Stinging nettle extract | 30 | 21.7-83.6 | 85 - 162 | 21 |
| **$Na_2SeO_3$** | *Terminalia arjuna* extract | 30 | 10-80 | - | 22 |
| **$H_2SeO_3$** | *Nilgirianthus ciliates* | 30±2 | >500 | 100-1000 | 23 |
| **$SeSO_4$** | Cardamomum extract | 35 | 38.26 - 68.49 | - | 24 |
| **$Na_2SeO_3$** | *Peltophorum pterocarpum* extract | 36 | 21-42 | - | 25 |
| **$Na_2SeO_3$** | *Catharanthus roseus(L.)* extract | 36 | 17-34 | - | 25 |

**Table 2: Influence of temperature on SeNp size**

| Precursor Solution | Plant Extract | Temperature (°C) | Core Size (nm) | Hydrodynamic diameter (nm) | References |
|---|---|---|---|---|---|
| $Na_2SeO_4$ | *Bougainvillea spectabilis* | 36 | 18-35 | - | [26] |
| $H_2SeO_3$ | *C. bulbosa* tuber extract | 37 | 50-200 | 55.9 | [27] |
| $Na_2SeO_3$ | *Artemisia annua* | 37 | 110-200 | 70.81 | [28] |
| $Na_2SeO_3$ | *Citrus reticulata* extract | 40 | 70 | - | [29] |
| $H_2SeO_3$ | *Spermacoce hispida* | 40 | 120 ± 15 | - | [30] |
| $Na_2SeO_3$ | *Cleome droserifolia* extract | 40 | 50-190 | 166.9 | [31] |
| $H_2SeO_3$ | *Cleistocalyx operculatus* leaf extract | 40 | 50-200 | - | [32] |
| $Na_2SeO_3$ | *Amphipterygium glaucum* extract | 40 | 40-60 | 142 | [33] |
| $Na_2SeO_3$ | *Luffa cylindrica* extract | 40 | 100 | - | [34] |
| $Na_2SeO_3$ | *Azadirachta indica* extract | 45 | 62.1 - 77.6 | - | [35] |
| $Na_2SeO_3$ | *Hibiscus esculentus* extract | 45-50 | 62 | - | [36] |
| $Na_2SeO_3$ | *Psidium guajava* extract | 60 | 8-20 | 70.93 | [37] |
| $Na_2SeO_3$ | *Glycosmis pentaphylla (Retz.) DC.* extract | 60 | 56.21 | 28-122 | [38] |
| $Na_2SeO_3$ | Propolis extract | 60 | 116.5 | - | [39] |
| $Na_2SeO_3$ | Onion peel extract | 60 | 100 | - | [40] |
| $Na_2SeO_3$ | *Allium sativum* extract | 70 | 40-100 | - | [41] |
| $Na_2SeO_3$ | Tomato fruit extract | 75 | 1155 | - | [42] |
| $Na_2SeO_3$ | Tomato seed extract | 75 | 1155 | - | [42] |
| $Na_2SeO_3$ | *Ocimum tenuiflorum L* extract | 80 | 64.23±1.1 | - | [43] |
| $Na_2SeO_3$ | *Cordia myxa* extract | 80 | 12 | - | [44] |
| $Na_2SeO_3$ | Xanthan gum | 80 | 44-68 | 65 | [45] |
| $Na_2SeO_3$ | *Ocimum gratissimum* extract | 90 | 20-100 | - | [46] |
| $H_2SeO_3$ | Lemon extract | 95 | 90 | - | [47] |
| $Na_2SeO_3$ | *Muntigia calabura* extract | 100 | 30-50 | - | [48] |
| $Na_2SeO_3$ | Coffee bin extract | 121 | 595 | - | [49] |
| $Na_2SeO_3$ | Garlic aqueous extract | 150 | - | 124±2 | [50] |

**Table 3: Zeta potential data and their variation with temperature.**

| Precursor Solution | Plant Extract | Temperature (°C) | Zeta-potential (mV) | References |
|---|---|---|---|---|
| **$Na_2SeO_3$** | *Moringa olefiera* extract | 25 | 1.94 | 51 |
| **$Na_2SeO_3$** | Hawthorn fruit extract | 25 | - 24.5 | 5 |
| **$Na_2SeO_3$** | Ginger fruit extract | 25 | -36 | 18 |
| **$H_2SeO_3$** | *Orthosiphon stamineus* extract | 25 | -34.9 | 9 |
| **$NaHSeO_3$** | *Petroselinum crispum* extract | 25 | -14.2 | 12 |
| **$H_2SeO_3$** | *Astericus graveolens* extract | 25 | -24.1 | 13 |
| **$H_2SeO_3$** | *Diospyros montana* leaf extract | 25 | -22.3 | 15 |
| **$Na_2SeO_3$** | *Zingiber officinale* extract | 25 | -36 | 18 |
| **$Na_2SeO_3$** | *Allium sativum* extract | 25 | -23.8 | 52 |
| **$Na_2SeO_3$** | *Allium sativum* extract | 25 | -24.2 | 52 |
| **$Na_2SeO_3$** | *Abelmoschus esculentus* extract | 25 | -64 | 53 |
| **$Na_2SeO_3$** | Boldo leaves + Acreola pulp | 25 | -23.46± 0.40 | 54 |
| **$Na_2SeO_3$** | Onion extract + Acreola Pulp Extract | 25 | -23.93± 0.20 | 54 |
| **$Na_2SeO_3$** | Boldo Leaves + Acreola Pulp Extract | 25 | -25.13± 0.55 | 54 |
| **$Na_2SeO_3$** | Onion extract + Acreola Pulp Extract | 25 | -26.93± 0.32 | 54 |
| **$Na_2SeO_3$** | Cinnamon extract | 25 | -28.6 | 20 |
| **$Na_2SeO_3$** | *Annona muricata* extract | 25 | -26 | 55 |
| **$SeSO_4$** | *Ephedra aphylla* extract | 25 | -5.61 | 56 |
| **$Na_2SeO_3$** | *Vaccinium arctostaphylos (L.)* fruit extract | 25 | −12.44 ± 1.38 | 57 |
| **$Na_2SeO_3$** | Costus extract | 25 | -42.8 | 58 |

**Table 3: Zeta potential data and their variation with temperature.**

| **Precursor Solution** | Plant Extract | Temperature (°C) | Zeta-potential (mV) | References |
|---|---|---|---|---|
| | | | | |

**Table 3: Zeta potential data and their variation with temperature.**

| **Precursor Solution** | Plant Extract | Temperature (°C) | Zeta-potential (mV) | References |
|---|---|---|---|---|
| $H_2SeO_3$ | *Averrhoa carambola* extract | 25 | -23.20 | [59] |
| $H_2SeO_3$ | *Carica papaya* extract | 25 | -17.8 | [60] |
| $Na_2SeO_3$ | *Zingiber officinale* extract | 25 | -36 | [18] |
| $Na_2SeO_3$ | Green tea extract | 25 | -24.1 | [61] |
| $Na_2SeO_3$ | *Ulva fasciata* | 25 | -28.57 | [62] |
| $SeO_2$ | Tree gum | 25 | −39.9 ± 4.1 | [63] |
| $SeO_2$ | Stinging nettle extract | 30 | -14.5 | [21] |
| $H_2SeO_3$ | *Nilgirianthus ciliates* | 30±2 | -9.16 | [23] |
| $SeSO_4$ | Cardamomum extract | 35 | -11 | [24] |
| $H_2SeO_3$ | *C. bulbosa* tuber extract | 37 | -17.8 | [27] |
| $Na_2SeO_3$ | *Artemisia annua* | 37 | -26.6 | [28] |
| $Na_2SeO_3$ | *Luffa cylindrica* extract | 40 | -13.6 | [34] |
| $Na_2SeO_3$ | *Hibiscus esculentus* extract | 45-50 | -51.3 | [36] |
| $Na_2SeO_3$ | *Psidium guajava* Extract | 60 | +24.75 | [37] |
| $Na_2SeO_3$ | Propolis extract | 60 | -46.1 | [39] |
| $Na_2SeO_3$ | *Ocimum tenuiflorum L* extract | 80 | - 46.4 | [43] |
| $Na_2SeO_3$ | *Cordia myxa* extract | 80 | 3.9 | [44] |
| $Na_2SeO_3$ | Xanthan gum | 80 | -35.46 | [45] |
| $Na_2SeO_3$ | *Muntigia calabura* extract | 100 | - 20.0 | [48] |
| $Na_2SeO_3$ | Coffee bin extract | 121 | + 6.8 | [49] |

**Table 3: Zeta potential data and their variation with temperature.**

| **Precursor Solution** | Plant Extract | Temperature (°C) | Zeta-potential (mV) | References |
|---|---|---|---|---|
| **$Na_2SeO_3$** | *Aloe vera* leaf extract (ALE) | 121 | -18 | [1] |
| **$Na_2SeO_3$** | *Cannabis sativa* | 121 | -45 | [64] |
| **$Na_2SeO_3$** | Potato extract | 121 | -29.3 | [65] |
| **$Na_2SeO_3$** | Garlic aqueous extract | 150 | -28.9±0.4 | [50] |

**Table 4: Relationship between temperature and polydispersity index of green-synthesized SeNp**

| Precursor Solution | Plant Extract | Temperature (°C) | Polydispersity Index (PDI) | References |
|---|---|---|---|---|
| **$H_2SeO_3$** | *Vitis vinifera* (Raisin) extract | 25 | 0.212 | [66] |
| **$Na_2SeO_3$** | *Allium sativum* extract | 25 | 0.234 | [67] |
| **$Na_2SeO_3$** | *Ulva fasciata* | 25 | 0.41 | [62] |
| **$SeO_2$** | Tree gum | 25 | 0.39 | [63] |
| **$H_2SeO_3$** | *Astericus graveolens* extract | 25 | 1 | [13] |
| **$H_2SeO_3$** | *Tinospora cordifolia* extract | 25 | 0.389 | [68] |
| **$Na_2SeO_3$** | Boldo leaves + Acreola pulp extract | 25 | 0.27±0.03 | [54] |
| **$Na_2SeO_3$** | Onion extract + Acreola pulp extract | 25 | 0.42±0.03 | [54] |
| **$Na_2SeO_3$** | Boldo leaves + Acreola pulp extract | 25 | 0.36±0.15 | [54] |
| **$Na_2SeO_3$** | Onion extract + Acreola pulp extract | 25 | 0.39±0.01 | [54] |
| **$H_2SeO_3$** | *Averrhoa carambola* extract | 25 | > 0.5 | [59] |
| **$H_2SeO_3$** | *Carica papaya* extract | 25 | 0.03 | [60] |
| **$Na_2SeO_3$** | Green tea extract | 25 | 0.241 | [61] |
| **$Na_2SeO_3$** | *Vaccinium arctostaphylos (L.)* fruit extract | 25 | 0.4 | [57] |
| **$Na_2SeO_3$** | *Emblica officinalis* fruit extract | 27 (±)2 | < 0.2 | [19] |
| **$Na_2SeO_3$** | Pomegranate peel extract | 30 | 0.321 | [69] |
| **$H_2SeO_3$** | *C. bulbosa* tuber extract | 37 | 0.03 | [27] |
| **$Na_2SeO_3$** | *Artemisia annua* | 37 | 0.147 | [28] |
| **$Na_2SeO_3$** | *Cleome droserifolia* extract | 40 | 0.392 | [31] |
| **$Na_2SeO_3$** | *Luffa cylindrica* extract | 40 | 0.291 | [34] |
| **$Na_2SeO_3$** | *Blumia axiliaris* stem extract | 50 | 0.035 | [70] |
| **$Na_2SeO_3$** | *Blumia axiliaris* root extract | 50 | 0.61 | [70] |

**Table 4: Relationship between temperature and polydispersity index of green-synthesized SeNp**

| **Precursor Solution** | Plant Extract | Temperature (°C) | Polydispersity Index (PDI) | References |
|---|---|---|---|---|
| | | | | |
| $Na_2SeO_3$ | *Psidium guajava* extract | 60 | 0.34 | [37] |
| $Na_2SeO_3$ | *Glycosmis pentaphylla (Retz.) DC.* Extract | 60 | 0.06 | [38] |
| $Na_2SeO_3$ | Propolis extract | 60 | 0.251 | [39] |
| $Na_2SeO_3$ | Tomato fruit extract | 75 | 0.432 | [42] |
| $Na_2SeO_3$ | Tomato seed extract | 75 | 0.761 | [42] |
| $Na_2SeO_3$ | *Ocimum tenuiflorum L* extract | 80 | 0.000083 | [43] |
| $Na_2SeO_3$ | *Cordia myxa* extract | 80 | 0.197 | [44] |
| $Na_2SeO_3$ | Xanthan gum | 80 | 0.12 | [45] |
| $Na_2SeO_3$ | Coffee bin extract | 121 | 0.748 | [49] |
| $Na_2SeO_3$ | *Aloe vera* leaf extract | 121 | 0.344 | [1] |
| $Na_2SeO_3$ | *Cannabis sativa* | 121 | 0.032 | [64] |
| $Na_2SeO_3$ | Garlic aqueous extract | 150 | 0.155 ±0.02 | [50] |

**Table 5: Influence of stirring speed on the particle size of green synthesized SeNp.**

| **Precursor Solution** | Plant Extract | Stirring speed (rpm) | Core size (nm) | Hydrodynamic diameter (nm) | References |
|---|---|---|---|---|---|
| **$Na_2SeO_3$** | *Annona muricata* extract | 50-100 | 80-120 | 120-160 | 55 |
| **$Na_2SeO_3$** | *Emblica officinalis* fruit extract | 120 | 15-40 | 20-60 | 19 |
| **$Na_2SeO_3$** | *Zingiber officinale* extract | 130 | 100 – 150 | 100 – 120 | 18 |
| **$Na_2SeO_3$** | Ginger fruit extract | 130 | 100-150 | - | 18 |
| **$Na_2SeO_3$** | *Allium sativum* extract | 130 | 21-40 | - | 52 |
| **$Na_2SeO_3$** | *Allium sativum* extract | 130 | 41-50 | - | 52 |
| **$Na_2SeO_3$** | Pomegranate peel extract | 150 | 10.4-31.5 | 22.4 | 69 |
| **$Na_2SeO_3$** | *Allium sativum* extract | 150 | 40-100 | - | 41 |
| **$SeO_2$** | Stinging nettle extract | 150 | 21.7-83.6 | 85 - 162 | 21 |
| **$Na_2SeO_3$** | Lemon leaf extract (LLE) | 200 | 60-80 | - | 4 |
| **$Na_2SeO_3$** | *Terminalia arjuna* extract | 200 | 10-80 | - | 22 |
| **$Na_2SeO_3$** | Tomato fruit extract | 200 | 1020 | - | 42 |
| **$Na_2SeO_3$** | Tomato seed extract | 200 | 1155 | - | 42 |
| **$Na_2SeO_3$** | Garlic aqueous extract | 200 | - | 124±2 | 50 |
| **$Na_2SeO_3$** | *Clitoria ternatea* extract | 250 | 11-106 | - | 71 |
| **$Na_2SeO_3$** | *Catharanthus roseus(L.)* extract | 250 | 17-34 | - | 25 |
| **$Na_2SeO_3$** | *Peltophorum pterocarpum* extract | 250 | 21-42 | - | 25 |
| **$Na_2SeO_3$** | Onion peel extract | 260 | 100 | - | 40 |
| **$Na_2SeO_3$** | Propolis extract | 300 | 116.5 | - | 39 |
| **$Na_2SeO_3$** | *Abelmoschus esculentus* extract | 400 | 17.3 | 46.5 | 53 |
| **$Na_2SeO_3$** | *Rosmarinus officinalis* extract | 400 | 39 | 7.9 | 72 |
| **$Na_2SeO_3$** | *Hibiscus esculentus* extract | 500 | 62 | - | 36 |

**Table 5: Influence of stirring speed on the particle size of green synthesized SeNp.**

| **Precursor Solution** | Plant Extract | Stirring speed (rpm) | Core size (nm) | Hydrodynamic diameter (nm) | References |
|---|---|---|---|---|---|
| $Na_2SeO_3$ | *Morinda citrifolia* leaf extract | 650-700 | 12-160 | - | [73] |
| $Na_2SeO_3$ | *Brassica oleracea* extract | 650 - 800 | 10 - 25 | - | [74] |
| $H_2SeO_3$ | Lemon extract | 800 | 90 | - | [47] |
| $Na_2SeO_3$ | *Amphipterygium glaucum* extract | 1200 | 40-60 | 142 | [33] |
| $Na_2SeO_3$ | Costus extract | 1268 | 2.86-8.73 | - | [58] |
| $Na_2SeO_3$ | Cinnamon extract | 1369 | 6.8 – 58.2 | - | [75] |

**Table 6: Correlation between stirring rate and zeta-potential of green synthesized SeNp**

| **Precursor Solution** | Plant Extract | Stirring speed (rpm) | Zeta Potential (mV) | References |
|---|---|---|---|---|
| **$Na_2SeO_3$** | *Annona muricata* extract | 50-100 | -26 | [55] |
| **$Na_2SeO_3$** | *Allium sativum* extract | 130 | -23.8 | [52] |
| **$Na_2SeO_3$** | *Allium sativum* extract | 130 | -24.2 | [52] |
| **$Na_2SeO_3$** | *Zingiber officinale* extract | 130 | -36 | [18] |
| **$Na_2SeO_3$** | Ginger fruit extract | 130 | -36 | [18] |
| **$SeO_2$** | Stinging nettle extract | 150 | -14.5 | [21] |
| **$Na_2SeO_3$** | Garlic aqueous extract | 200 | -28.9±0.4 | [50] |
| **$Na_2SeO_3$** | Propolis extract | 300 | - 46.1 | [39] |
| **$Na_2SeO_3$** | *Abelmoschus esculentus* extract | 400 | -64 | [53] |
| **$Na_2SeO_3$** | *Rosmarinus officinalis* extract | 400 | -78 | [72] |
| **$Na_2SeO_3$** | *Hibiscus esculentus* extract | 500 | -51.3 | [36] |
| **$Na_2SeO_3$** | Costus extract | 1268 | -42.8 | [58] |
| **$Na_2SeO_3$** | Cinnamon extract | 1369 | -28.6 | [75] |

**Table 7: Correlation between stirring speed and polydispersity index (PDI)**

| **Precursor Solution** | Plant Extract | Stirring speed (rpm) | Polydispersity index (PDI) | References |
|---|---|---|---|---|
| **$Na_2SeO_3$** | *Emblica officinalis* fruit extract | 120 | < 0.2 | [91] |
| **$Na_2SeO_3$** | Pomegranate peel extract | 150 | 0.321 | [137] |
| **$Na_2SeO_3$** | Tomato fruit extract | 200 | 0.432 | [112] |
| **$Na_2SeO_3$** | Tomato seed extract | 200 | 0.761 | [112] |
| **$Na_2SeO_3$** | Garlic aqueous extract | 200 | 0.155 ±0.02 | [118] |
| **$Na_2SeO_3$** | Propolis extract | 300 | 0.251 | [109] |

# References


1 B. Fardsadegh and H. Jafarizadeh-Malmiri, *Green Processing and Synthesis*, 2019, 8, 399–407.

2 B. Fardsadegh, H. Vaghari, R. Mohammad-Jafari, Y. Najian and H. Jafarizadeh-Malmiri, *Green Processing and Synthesis*, 2019, 8, 191–198.

3 D. Fan, L. Li, Z. Li, Y. Zhang, X. Ma, L. Wu, H. Zhang and F. Guo, *Sci Technol Adv Mater*, 2020, 21, 505–514.

4 K. S. Prasad, H. Patel, T. Patel, K. Patel and K. Selvaraj, *Colloids Surf B Biointerfaces*, 2013, 103, 261–266.

5 D. Cui, T. Liang, L. Sun, L. Meng, C. Yang, L. Wang, T. Liang and Q. Li, *Pharm Biol*, 2018, 56, 528–534.

6 V. Alagesan and S. Venugopal, *Bionanoscience*, 2019, 9, 105–116.

7 CH. Ramamurthy, K. S. Sampath, P. Arunkumar, M. S. Kumar, V. Sujatha, K. Premkumar and C. Thirunavukkarasu, *Bioprocess Biosyst Eng*, 2013, 36, 1131–1139.

8 K. Anu, S. Devanesan, R. Prasanth, M. S. AlSalhi, S. Ajithkumar and G. Singaravelu, *J King Saud Univ Sci*, 2020, 32, 2520–2526.

9 C. Sivakumar and K. Jeganathan, *Journal of Drug Delivery and Therapeutics*, 2018, 8, 195–200.

10 A. S. S. B. R. Kirupagaran, *J. Nanosci. Tech. 2(5) (2016) 224–226.* , 2016, 224–226.

11 Jay Vyas and Shafkat Rana, *The Pharma Innovation Journal* , 2018, 7(9), 262–266.

12 V. L. S. C. T. C. S. I. V. Luminita Fritea1, *Studia Universitatis "Vasile Goldiş", Seria Ştiinţele Vieţii* , 2017, 27, 203–208.

13 S. Y. S. Zeebaree, A. Y. S. Zeebaree and O. I. H. Zebari, *Sustain Chem Pharm*, 2020, 15, 100210.

14 P. Sowndarya, G. Ramkumar and M. S. Shivakumar, *Artif Cells Nanomed Biotechnol*, 2017, 45, 1490–1495.

15 K. Kokila, N. Elavarasan and V. Sujatha, *New Journal of Chemistry*, 2017, 41, 7481–7490.

16 R. M. Tripathi, P. Hameed, R. P. Rao, N. Shrivastava, J. Mittal and S. Mohapatra, *Bionanoscience*, 2020, 10, 389–396.

17 R. Hassanien, A. A. I. Abed-Elmageed and D. Z. Husein, *ChemistrySelect*, 2019, 4, 9018–9026.

18 S. Menon, S. D. K.S., H. Agarwal and V. K. Shanmugam, *Colloid Interface Sci Commun*, 2019, 29, 1–8.

19 L. Gunti, R. S. Dass and N. K. Kalagatur, *Front Microbiol*, DOI:10.3389/fmicb.2019.00931.

20 N. M. AL-Roomi and H. H. Ajeel, *UTTAR PRADESH JOURNAL OF ZOOLOGY*, 2024, 45, 80–90.

21 A. H. Hashem and S. S. Salem, *Biotechnol J*, DOI:10.1002/biot.202100432.

22 K. S. Prasad and K. Selvaraj, *Biol Trace Elem Res*, 2014, 157, 275–283.

23 K. Meenambigai, R. Kokila, K. Chandhirasekar, A. Thendralmanikandan, D. Kaliannan, K. S. Ibrahim, S. Kumar, W. Liu, B. Balasubramanian and A. Nareshkumar, *Biol Trace Elem Res*, 2022, 200, 2948–2962.

24 S. Zubair Dhabian and R. Sabeeh Jasim, *Journal of Nanostructures*, 2023, 13, 76–85.

25 B. Deepa and V. Ganesan*, *International Journal of ChemTech Research* , 2014, 7, 725–733.

26 B. G. V. Deepa, *International Journal of Science and Research*.

27 V. Cittrarasu, D. Kaliannan, K. Dharman, V. Maluventhen, M. Easwaran, W. C. Liu, B. Balasubramanian and M. Arumugam, *Sci Rep*, 2021, 11, 1032.

28 A. V. A. Mariadoss, K. Saravanakumar, A. Sathiyaseelan, K. V. Naveen and M.-H. Wang, *Microb Pathog*, 2022, 167, 105544.

29 Sowmiya. R. Balakrishnaraja. R. Santanu Sasidharan, *World Journal of Pharmaceutical Research* , 2014, 4, 1322–1330.

30 K. Vennila, L. Chitra, R. Balagurunathan and T. Palvannan, *Advances in Natural Sciences: Nanoscience and Nanotechnology*, 2018, 9, 015005.

31 H. A. Abdel Maksoud, O. A. R. Abou Zaid, M. G. Elharrif, M. A. Omnia and E. A. Alaa, *Clin Nutr ESPEN*, 2020, 40, 383–391.

32 T. T. Vu, P. T. M. Nguyen, N. H. Pham, T. H. Le, T. H. Nguyen, D. T. Do and D. D. La, *Journal of Composites Science*, 2022, 6, 307.

33 J. J. O. Garza-García, J. A. Hernández-Díaz, J. M. León-Morales, G. Velázquez-Juárez, A. Zamudio-Ojeda, J. Arratia-Quijada, O. K. Reyes-Maldonado, J. C. López-Velázquez and S. García-Morales, *J Nanobiotechnology*, 2023, 21, 252.

34 M. Soni, R. Gayathri, K. Sankaran, V. P. Veeraraghavan and A. P. Francis, *Nano*, DOI:10.1142/S179329202350042X.

35 M. A. Hawsah, R. Abdel-Gaber, S. Al-Quraishy, H. M. A. Aljawdah, S. N. Maodaa and E. Al-Shaebi, *Food Science and Technology*, DOI:10.5327/fst.13223.

36 M. A. Ebrahimzadeh, M. Moradsomarein, F. S. Lalerdi and S. R. Alizadeh, *European Journal of Chemistry*, 2023, 14, 144–154.

37 H. Alam, N. Khatoon, M. Raza, P. C. Ghosh and M. Sardar, *Bionanoscience*, 2019, 9, 96–104.

38 R. D. Sarkar, P. Lahkar and M. C. Kalita, *Bioresour Technol Rep*, 2022, 17, 100894.

39 R. Hatami, A. Javadi and H. Jafarizadeh-Malmiri, *Green Processing and Synthesis*, 2020, 9, 685–692.

40 F. Martínez-Esquivias, J. M. Guzmán-Flores and A. Perez-Larios, *Particulate Science and Technology*, 2023, 41, 319–329.

41 R. AFTAB, S. AHSAN, A. LIAQAT, M. SAFDAR, M. F. J. CHUGHTAI, M. NADEEM, M. A. FAROOQ, T. MEHMOOD and A. KHALIQ, *Food Science and Technology*, DOI:10.1590/fst.67022.

42 Sani-e-Zahra, M. S. Iqbal, K. Abbas and M. I. Qadir, *Arabian Journal of Chemistry*, 2022, 15, 103901.

43 F. Olawale, M. Ariatti and M. Singh, *Advances in Natural Sciences: Nanoscience and Nanotechnology*, 2022, 13, 015015.

44 L. Hosseinpour, J. Baharara, S. Zaker Bostanabad and M. Darroudi, *Inorg Chem Commun*, 2022, 145, 110030.

45 M. Velayati, H. Hassani, H. A. Hosseini, Z. Sabouri, A. Mostafapour and M. Darroudi, *The European Physical Journal Plus*, 2023, 138, 947.

46 G. E. Ogunleye, K. A. Oyinlola, O. Akintade, R. Fashogbon and T. Adesina, *Turkish Journal of Agriculture - Food Science and Technology*, 2022, 10, 2903–2912.

47 V. J. Sawant and V. J. Sawant, *Sens Biosensing Res*, 2020, 27, 100314.

48 N. N. K. Tuyen, V. K. Huy, N. H. Duy, H. An, N. T. H. Nam, N. M. Dat, Q. T. T. Huong, N. L. P. Trang, N. D. P. Anh, L. T. M. Thy, M. T. Phong and N. H. Hieu, *Waste Biomass Valorization*, 2024, 15, 1987–1998.

49 R. Abbasian and H. Jafarizadeh-Malmiri, *Open Agric*, 2020, 5, 761–767.

50 L. Mirzakhani, H. Jafarizadeh-Malmiri and O. Ahmadi, *Nano-Structures & Nano-Objects*, 2024, 38, 101162.

51 M. Banerjee and V. D. Rajeswari, *Biocatal Agric Biotechnol*, 2024, 55, 102978.

52 N. J. K. J. jirasripongpun_k@su. ac. th Pinprapha Sribenjarat, *Science, Engineering and Health Studies*, 2020, 14, 22–31.

53 R. S. Ghaderi, F. Adibian, Z. Sabouri, J. Davoodi, M. Kazemi, S. Amel Jamehdar, Z. Meshkat, S. Soleimanpour and M. Daroudi, *Materials Technology*, 2022, 37, 1289–1297.

54 L. M. dos S. Souza, M. Dibo, J. J. P. Sarmiento, A. B. Seabra, L. P. Medeiros, I. M. Lourenço, R. K. T. Kobayashi and G. Nakazato, *Current Research in Green and Sustainable Chemistry*, 2022, 5, 100303.

55 C. Rao, U. Mangamuri, A. Sikharam, K. Devaraj, N. Kalagatur and K. Kadirvelu, *Curr Trends Biotechnol Pharm*, 2022, 16, 101–107.

56 M. M. El-Zayat, M. M. Eraqi, H. Alrefai, A. Y. El-Khateeb, M. A. Ibrahim, H. M. Aljohani, M. M. Aljohani and M. M. Elshaer, *Biomolecules*, 2021, 11, 470.

57 M. A. A. Khudier, H. A. Hammadi, H. T. Atyia, H. Al-Karagoly, S. Albukhaty, G. M. Sulaiman, Y. H. Dewir and H. B. Mahood, *Cogent Food Agric*, DOI:10.1080/23311932.2023.2245612.

58 M. S. Al-Saggaf, A. A. Tayel, M. O. I. Ghobashy, M. A. Alotaibi, M. A. Alghuthaymi and S. H. Moussa, *Green Processing and Synthesis*, 2020, 9, 477–487.

59 M. P. Ganeshkar, M. R. Mirjankar, P. Shivappa, A. T. Gaddigal, P. H. Goder and C. M. Kamanavalli, *Particulate Science and Technology*, 2023, 41, 990–1002.

60 S. Rajasekar and S. Kuppusamy, *J Clust Sci*, 2021, 32, 907–915.

61 W. Zhang, J. Zhang, D. Ding, L. Zhang, L. A. Muehlmann, S. Deng, X. Wang, W. Li and W. Zhang, *Artif Cells Nanomed Biotechnol*, 2018, 46, 1463–1470.

62 K. Shahzamani, H. E. Lashgarian, M. Karkhane, A. Ghaffarizadeh, S. Ghotekar and A. Marzban, *Emergent Mater*, 2022, 5, 1689–1698.

63 A. J. Kora, *IET Nanobiotechnol*, 2018, 12, 658–662.

64 H. Mehta, M. Gupta, P. Kaur, J. Kaur and N. Kaushal, *Applied Science and Convergence Technology*, 2021, 30, 81–86.

65 S. Chandramohan, K. Sundar and A. Muthukumaran, *IET Nanobiotechnol*, 2019, 13, 275–281.

66 G. Sharma, A. Sharma, R. Bhavesh, J. Park, B. Ganbold, J.-S. Nam and S.-S. Lee, *Molecules*, 2014, 19, 2761–2770.

67 P. B. Ezhuthupurakkal, L. R. Polaki, A. Suyavaran, A. Subastri, V. Sujatha and C. Thirunavukkarasu, *Materials Science and Engineering: C*, 2017, 74, 597–608.

68 P. S. Puri A, *Pharmacog Res.* , 2022, 14, 1–7.

69 A. H. Hashem, E. Saied, O. M. Ali, S. Selim, S. K. Al Jaouni, F. M. Elkady and G. S. El-Sayyad, *Appl Biochem Biotechnol*, 2023, 195, 5753–5776.

70 J. P. Dash, L. Mani and S. K. Nayak, *Egyptian Journal of Basic and Applied Sciences*, 2022, 9, 65–76.

71 M. D. Barma and S. Doraikanan, *Int J Health Sci (Qassim)*, 2022, 2529–2538.

72 F. Adibian, R. S. Ghaderi, Z. Sabouri, J. Davoodi, M. Kazemi, K. Ghazvini, M. Youssefi, S. Soleimanpour and M. Darroudi, *BioMetals*, 2022, 35, 147–158.

73 M. Nagalingam, S. Rajeshkumar, S. K. Balu, M. Tharani and K. Arunachalam, *J Nanomater*, DOI:10.1155/2022/2155772.

74 G. Dhanraj and S. Rajeshkumar, *J Nanomater*, 2021, 2021, 1–9.

75 M. A. Alghuthaymi, A. M. Diab, A. F. Elzahy, K. E. Mazrou, A. A. Tayel and S. H. Moussa, *J Food Qual*, 2021, 2021, 1–10.
